# CREPT Disarms the Inhibitory Activity of HDAC1 on Oncogene Expression to Promote Tumorigenesis

**DOI:** 10.3390/cancers14194797

**Published:** 2022-09-30

**Authors:** Yajun Cao, Bobin Ning, Ye Tian, Tingwei Lan, Yunxiang Chu, Fangli Ren, Yinyin Wang, Qingyu Meng, Jun Li, Baoqing Jia, Zhijie Chang

**Affiliations:** 1 State Key Laboratory of Membrane Biology, School of Medicine, Tsinghua University, Beijing 100084, China; 2 Department of General Surgery, The First Medical Centre, Chinese PLA General Hospital, Beijing 100039, China; 3 Department of Gastroenterology, Emergency General Hospital, Beijing 100028, China; 4 Qingda Cell Biotech Inc., Beijing 100084, China

**Keywords:** CREPT, HDAC1, tumorigenesis, Wnt signaling, oncogene, tumor suppressor genes

## Abstract

**Simple Summary:**

It has been proposed that highly expressed HDAC1 (histone deacetylases 1) removes the acetyl group from the histones at the promoter regions of tumor suppressor genes to block their expression in tumors. We here revealed the underlying mechanism that HDAC1 differentially regulates the expression of oncogenes and tumor suppressors. In detail, we found that HDAC1 is unable to occupy the promoters of oncogenes but maintains its occupancy with the tumor suppressors due to its interaction with an oncoprotein, CREPT (cell cycle-related and expression-elevated protein in tumor).

**Abstract:**

Histone deacetylases 1 (HDAC1), an enzyme that functions to remove acetyl molecules from ε-NH3 groups of lysine in histones, eliminates the histone acetylation at the promoter regions of tumor suppressor genes to block their expression during tumorigenesis. However, it remains unclear why HDAC1 fails to impair oncogene expression. Here we report that HDAC1 is unable to occupy at the promoters of oncogenes but maintains its occupancy with the tumor suppressors due to its interaction with CREPT (cell cycle-related and expression-elevated protein in tumor, also named RPRD1B), an oncoprotein highly expressed in tumors. We observed that CREPT competed with HDAC1 for binding to oncogene (such as *CCND1*, *CLDN1*, *VEGFA*, *PPARD* and *BMP4*) promoters but not the tumor suppressor gene (such as p21 and p27) promoters by a chromatin immunoprecipitation (ChIP) qPCR experiment. Using immunoprecipitation experiments, we deciphered that CREPT specifically occupied at the oncogene promoter via TCF4, a transcription factor activated by Wnt signaling. In addition, we performed a real-time quantitative PCR (qRT-PCR) analysis on cells that stably over-expressed CREPT and/or HDAC1, and we propose that HDAC1 inhibits CREPT to activate oncogene expression under Wnt signaling activation. Our findings revealed that HDAC1 functions differentially on tumor suppressors and oncogenes due to its interaction with the oncoprotein CREPT.

## 1. Introduction

Tumorigenesis occurs due to the dysregulation of oncogenes and tumor suppressors [1]. To sustain growth and eventually progress to metastasis, tumors undergo several molecular alterations including gene mutations, epigenetic rewiring and the preferable transcription of specific genes [2,3]. Among these alterations, epigenetic rewiring has been recognized to regulate gene expression via modifying histones. These include histone acetylation, methylation, phosphorylation, ADP-ribosylation, sumoylation, and ubiquitylation [4,5]. To date, histone acetylation is reported to regulate gene expression by opening gene promoters at the chromatin [6,7]. In general, histone acetylation is mediated by two opposing sets of enzymes: HATs (histone acetyltransferases) and HDACs (histone deacetylases). While HATs induce histone acetylation by adding the acetyl group to the ε-NH+ of lysine residues, HDACs remove the acetyl group to maintain histones, as well as non-histone proteins, in a deacetylated status. Totally, 18 HDACs were identified in humans, and divided into four sub-classes according to their structural similarities: class I HDACs (HDAC1, 2, 3, and 8), class IIa (HDAC4, 5, 7, and 9), class IIb (HDAC6, 10), class III (Sirt1–7), and class IV (HDAC11) [8].

Numerous studies have revealed that HDACs are abnormally expressed in human tumors [9]. In particular, HDAC1 is elevated in a variety of cancers, including gastric, colorectal, lung, and bladder cancers [10,11,12,13,14]. It appears that the high level of HDAC1 promotes tumorigenesis, therefore, the inhibition of HDAC1 induces cell cycle arrest, decreases viability, and increases apoptosis in cancer cells [13,15,16,17,18]. The molecular mechanism for abundantly expressed HDAC1 in promoting tumor cell growth was attributed to the repression of tumor suppressor genes [19,20,21,22,23]. HDAC1 was characterized to occupy and deacetylate histones at the promoters of tumor suppressor genes to silence their expression. Therefore, the inhibition of HDCA1, as well as other HDACs, has been widely used as a target for cancer therapy by using small molecule inhibitors. However, it remains a question whether highly expressed HDAC1 represses oncogene expression during tumorigenesis. Indeed, several studies reported that HDAC1 represses Wnt signaling, an evolutionarily conserved pathway [24], in the regulation of cell proliferation, cell polarity, and cell fate determination during embryonic and cancer progression [25].

Wnt signaling activates oncogene expression through the transcription factors TCF/LEF (T-cell factor/lymphoid enhancer factor), which bind to a conservative DNA sequence CCTTTGWW (W represents either T or A) [26]. In the absence of Wnt signaling, HDAC1 associates with Transducin-like enhancer/Groucho (TLE/GRG) to repress TCF activation. HDAC1 silences chromatin by maintaining oncogene promoters in a deacetylated status. Upon Wnt stimulation, β-catenin is accumulated in the nucleus to displace Groucho/HDACs and recruits other co-activators for gene transcription [26]. In this context, the high level of HDAC1 functions to repress Wnt-activated oncogene expression. The inhibitory role of HDAC1 on tumor suppressors and oncogenes appears to be paradoxical for tumors, as tumor cells express high levels of oncogenes and low levels of tumor suppressors. Therefore, tumor cells must have a mechanism to disable the inhibitory ability of HDAC1 for oncogenes and to allow HDAC1 to inhibit only tumor suppressors.

CREPT (cell cycle-related and expression elevated protein in tumor) is an oncoprotein highly expressed in tumor tissues [27,28,29,30]. Our group recently revealed that CREPT promotes the transcription of Wnt/β-catenin signaling target genes by enhancing β-catenin/TCF4 interactions and promoting p300-mediated β-catenin acetylation [31,32]. On the other hand, we found that p15RS, an orthologue of CREPT, was associated with HDAC2 and represses gene expression [33]. Here, we demonstrate that elevated CREPT prevents HDAC1 from binding to the promoters of oncogenes and increases the level of acetylated histone H3 to facilitate oncogene transcription in tumorigenesis. We propose that abundantly expressed CREPT disarms the repression function of HDAC1 as regards the oncogene transcription but not the tumor suppressor gene expression.

## 2. Materials and Methods

### 2.1. Cell Culture and Transfections

HEK293T, SW480, SW620, and HCT116 cells were cultured in DMEM supplemented with 10% fetal bovine serum (FBS) and 1% penicillin/streptomycin (P/S). NCM460 and DLD1 were maintained in RPMI-1640 supplemented with 10% FBS and 1% P/S. Lovo was maintained in F12K supplemented with 10% FBS and 1% P/S. Cells were grown at 37 °C in a 5% CO_2_ humidified incubator. Cells were transfected with plasmids or siRNAs as indicated using Vigofect (Vigorous Inc., Punjab, Pakistan) or Lipofectamine RNAiMAX (Thermo Fisher, Waltham, MA, USA). To generate the stable cell line, cells were infected by lentivirus, which was produced by HEK293T cells. Database Name: National Infrastructure of Cell Line Resource, Accession Numbers: 1101HUM-PUMC000166 SW480; 1101HUM-PUMC000164 LoVo; 1101HUM-PUMC000091 293T; 1101HUM-PUMC000671 DLD-1; 1101HUM-PUMC000158 HCT 116; 1101HUM-PUMC000207 SW620; 1101HUM-PUMC000013 MCF7; 1101HUM-PUMC000406 MCF-10A; 1101HUM-PUMC000265 T47D; 1101HUM-PUMC000014 MDA-MB-231. NCM460 was kindly provided by Dr. Ye-Guang Chen, Tsinghua University.

### 2.2. Plasmids and Reagents

Myc-CREPT, HA–HDAC1, HA–HDAC2, HA–HDAC3, HA–TCF4, Flag–β-catenin, and SuperTop-luciferase were constructed in this laboratory. Anti-HDAC2 (Santa Cruz, Santa Cruz, CA, USA, H54), anti-HA (Santa Cruz, F-7), anti-Myc (Santa Cruz, 9E10), anti-HDAC1 (CST, Danvers, MA, USA, 34589, 5356; Abcam, Cambridge, UK, ab19845), anti-HDAC3 (CST, 3949; Abcam, ab137704), anti-TCF4 (CST, 2569), anti-H3K27ac (Abcam, ab4729), anti-actin (Sigma, St. Louis, MI, USA, AC-15), anti-Flag (Sigma, M2), and anti-β-catenin (BD, Franklin Lakes, NJ, USA, 4171778) were used. We generated an anti-CREPT antibody in our laboratory [34]. Short interfering RNAs (siRNAs) against HDAC1 (siHDAC1-1: 5′-GCCGGUCAUGUCCAAAGUATT-3′; siHDAC1-2: 5′-GCGACUGUUUGAGAACCUUTT-3′) were used. The guider RNAs sequences for deleting CREPT were 5′-CGGTGCCACACGGAGACGAT-3′ and 5′-GCTAAGCCCCCTGTGACGTT-3′.

### 2.3. Human Tumor Specimens and Staining

Human tumor specimens were acquired from the Chinese PLA General Hospital and then stained with different antibodies. The tissue collection procedure with informed consent was approved by the Clinical Ethics Committee of the Chinese PLA General Hospital.

### 2.4. Co-Immunoprecipitation and Western Blotting

For exogenous interaction assays, cells were transfected with the indicated plasmids. For endogenous interaction assays, cell lysates were collected by cell lysis buffer (50 mM Tris–Cl, 150 mM NaCl, 1% NP40, 0.5% sodium deoxycholate, 1% SDS, pH 8.0 and protease inhibitors). Then, cell lysates were incubated with antibodies and protein G-Sepharose beads (Cytiva, Marlborough, MA, USA) overnight at 4 °C. Beads were washed four times with cell lysis buffer. Precipitates were eluted and analyzed by Western blotting with the indicated antibodies.

For re-IP assays, precipitates were eluted by the 3 × Flag peptide. The precipitates were incubated with other antibodies and protein G-Sepharose beads at 4 °C for 16 h. Then, we washed the beads 4 times and eluted the re-precipitates for analysis.

### 2.5. Immunofluorescence Analysis

Cells were transfected with the indicated plasmids. Twenty-four hours later, cells were fixed by 4% paraformaldehyde solution and permeabilized by 0.3% Triton X-100. Cells were incubated in 10% FBS for 1 h and overnight with the antibodies at 4 °C. The secondary antibodies conjugated with FITC or TRITC (Jackson Research Laboratories) were added to cells for 1 h. Then, DAPI was added to the cells for 10 min. Lastly, the cells were imaged by laser scanning confocal microscopy (OLYMPUS FV3000).

### 2.6. Colony Formation Assay and Cell Viability Assay

For colony formation assay, 1 × 10^3^ indicated cells were seeded into one well in 6-well plate. All cells were repeated in three wells. After culturing for two weeks, washed cells with PBS. Then stained cells with 0.1% crystal violet for 1 h at room temperature. ImageJ was used to count the number of colonies.

For cell viability assay, 1 × 10^3^ indicated cells were seeded into one well in 96-well plates and cultured for indicated days. All cells were repeated in three wells. Then discarded the medium and added the CCK-8 kit reagent (Solarbio, Beijing, China) to detect the value of OD450 by spectrophotometer at indicated days.

### 2.7. Luciferase Assays

SuperTOP-Luc reporter, pRL-TK, and other indicated plasmids were co-transfected in HEK293T cells. After 4 h, Wnt3a condition medium was added to cells overnight. Then, we collected the cells and analyzed the luciferase signal by the Dual-Luciferase Assay System (Vigorous Inc.).

### 2.8. Real-Time Quantitative PCR (qRT-PCR) Analysis

RNAs were extracted using TRIzol (Invitrogen, Waltham, MA, USA) and cDNAs were obtained using a Quantscript RT Kit (TIANGEN Biotech, Beijing, China). The Talent qPCR PreMix Kit (SYBR Green, Tiangen Biotech) was used for qRT-PCR according to the manufacturer’s protocol. The sequences of the primers are shown in Supplemental List S1.

### 2.9. Chromatin Immunoprecipitation (ChIP) Assay

Cells were fixed in 1% formaldehyde solution at 37 °C for 10 min. Chromatin was sheared into 100–500 bp fragments by sonication at 4 °C. Then, the indicated antibodies were added into chromatin for ChIP analyses. Anti-IgG antibodies were used as a negative control. ChIPed DNA was extracted by phenol/chloroform and used for qPCR analysis. The sequences of primers are shown in Supplemental List S1.

### 2.10. Experimental Animals

Female BALB/c mice, 6 weeks old, were housed in isolated ventilated cages (five mice per cage) in a barrier facility at Tsinghua University with controlled environmental parameters: 22–26 °C, 12/12 h light/ dark cycle, sterile pellet food, and water ad libitum. The laboratory animal facility has been accredited by the AAALAC (Association for Assessment and Accreditation of Laboratory Animal Care International) and the IACUC (Institutional Animal Care and Use Committee) of Tsinghua University, which approved all animal protocols used in this study.

### 2.11. Tumor Formation

A total of 1 × 10^6^ indicated cells were subcutaneously injected into both sides of the mice. Then, the mice were sacrificed after 4 weeks. The volume and mass of tumors were measured.

### 2.12. Statistical Analysis

All experiments were repeated at least 3 times. GraphPad Prism was used for statistical analysis. Data are presented as mean ± standard deviation. Significant differences between groups were determined using a two-tailed unpaired Student’s *t*-test. *** *p* < 0.001, ** *p* < 0.01, * *p* < 0.05.

## 3. Results

### 3.1. CREPT Is Positively Correlated with HDAC1 in Human Tumors

**Figure 1:**
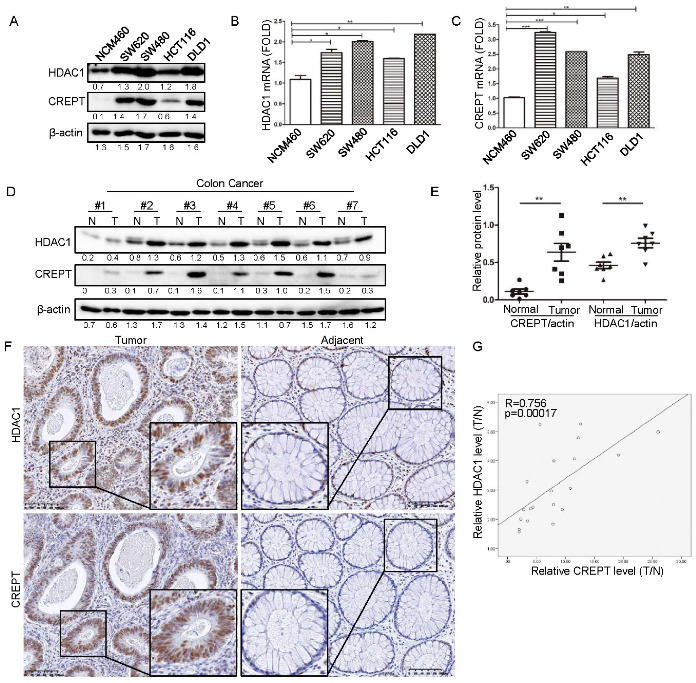
The correlation of HDAC1 and CREPT in human tumors. (**A**). CREPT expression is positively correlated to HDAC1 at the protein level in colon cancer cells. The endogenous protein levels of HDAC1 and CREPT in colon cancer cell lines (SW620, SW480, HCT116, DLD1) and epithelial cells (NCM460) are shown. β-actin was used as a loading control. (**B**,**C**). Both HDAC1 (**B**) and CREPT (**C**) are highly expressed in colon cancer cells when compared with the normal cells at the mRNA level. The mRNA levels were normalized to the fold change relative to β-actin. (**D**). Both HDAC1 and CREPT are elevated in the tumor tissues compared with the adjacent normal tissues from human colon cancers. Western blot analyses were based on β-actin as a loading control. N represents paired adjacent normal tissue and T represents tumor tissue from the same patient. The bands were quantified using ImageJ and presented as a value normalized according to the moderate level of a band in each blot. (**E**). The relative level of CREPT and HDAC1 in adjacent normal and tumor tissues are shown. The ratio of CREPT or HDAC1 vs actin was calculated by quantifying the bands from the Western blot in (**D**). The closed circle represents normal tissue for CREPT/actin, the square represents tumor tissue for CREPT/actin, the triangle represents normal tissue for HDAC1/actin and the inverted triangle represents tumor tissue for HDAC1/actin. (**F**). HDAC1 and CREPT were co-stained in human colon tumor tissue. Immunohistochemical staining was performed with an antibody against CREPT or HDAC1. (**G**). A graphical presentation of correlation of CREPT and HDAC1 in colon cancer. The levels of CREPT and HDAC1 in tumor tissue and the adjacent normal tissue were quantified using Image J. The ratio of CREPT (*X*-axis) or HDAC1 (*Y*-axis) level in tumor tissue to the adjacent normal tissue was calculated. SPSS was employed to figure out the correlation coefficient between the two proteins. (* *p* < 0.05, ** *p* < 0.01, *** *p* < 0.001).

Previous studies showed that CREPT or HDAC1 are highly expressed in tumors. However, whether CREPT and HDAC1 expression correlate remains to be demonstrated [27,35,36]. To analyze the potential relevance of CREPT and HDAC1, we examined their expression in different cell lines and human tissues. Western blot analyses showed that both HDAC1 and CREPT are highly expressed in all colon cancer cells including SW620, SW480, HCT116, and DLD1, but are either not or minimally expressed in the normal colon epithelial cell (NCM460) (Figure 1A). Real-time quantitative PCR (qRT-PCR) analyses consistently revealed that the mRNA levels of CREPT and HDAC1 were elevated in the cancer cells when compared with the normal epithelial cells (Figure 1B,C). The correlated expression of CREPT and HDAC1 was also observed in other cancers including breast, lung, and prostate cancer (Supplementary Figure S1). Another Western blot analysis on human colon cancer tissues demonstrated that both HDAC1 and CREPT were highly expressed in the tumor tissues in comparison with the paired adjacent tissues (Figure 1D,E). Immunohistochemistry analyses showed that both CREPT and HDAC1 were strongly stained in colon cancer tissues but weakly stained in the adjacent tissues (Figure 1F). The relative expressions of CREPT and HDAC1 in cancer tissue compared to the normal tissue were significantly correlated (R = 0.756, *p* = 0.00017) (Figure 1G). Generally, these results confirmed that CREPT and HDAC1 are coincidently elevated in colon cancer cells, raising a question that HDAC1 and CREPT might interplay to regulate tumor cell behaviors.

### 3.2. HDAC1 Represses the Activity of CREPT in Promoting Tumorigenesis

**Figure 2:**
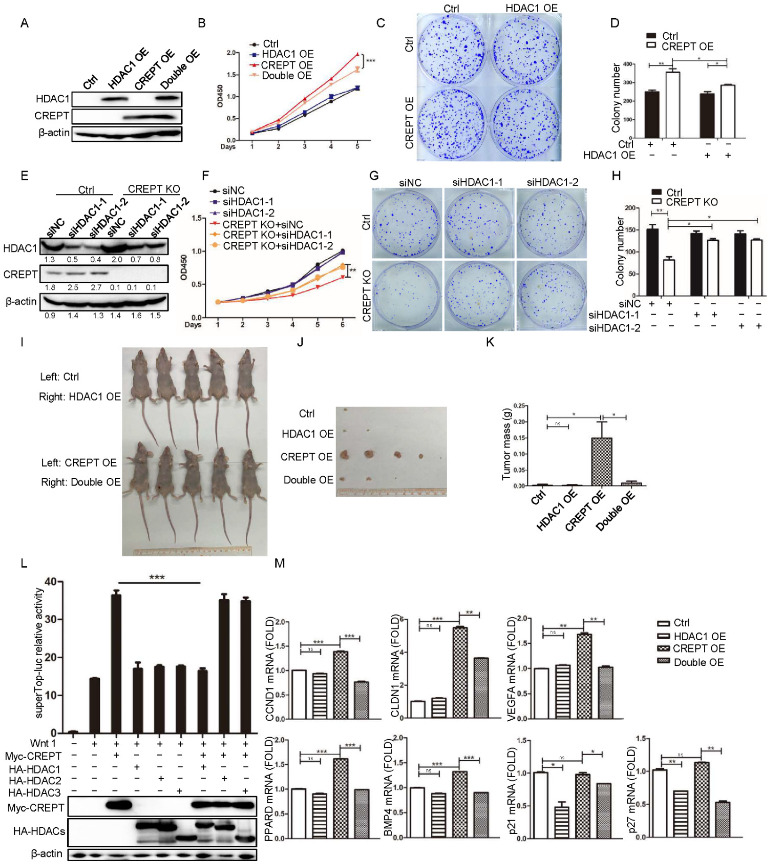
HDAC1 represses the activity of CREPT in promoting tumorigenesis. Stable cell lines were established for the overexpression of HDAC1 (HDAC1 OE) alone, CREPT (CREPT OE) alone, and both HDAC1 and CREPT (HDAC1&CREPT OE) in DLD1 cells, as well as for the deletion of HDAC1 (siHDAC1) or deletion of CREPT (CREPT KO) in SW480 cells. An empty vector was used as a control (EV) for overexpression experiments in DLD1 cells and a non-specific siRNA (siNC) was used as a control for deletion experiments in SW480 cells. Indicated stable cell lines were cultured for the CCK8 experiment at different days. A colony formation experiment was performed. A statistic result for the colony numbers as scanned by Image J is presented on the right panel. (**A**–**D**). Ectopic expression of HDAC1 significantly inhibits cell proliferation (**A**,**B**) or colony formation (**C**,**D**) in the presence of overexpressed CREPT in DLD1 cells. (**E**–**H**). Knocking down the expression of HDAC1 elevates cell proliferation (**E**,**F**) or colony formation (**G**,**H**) in the absence of CREPT in SW480 cells. The bands were quantified using ImageJ and presented as a value normalized according to the moderate level of a band in each blot. (**I**–**K**). Overexpression of HDAC1 inhibits the tumor formation in the presence of overexpressed CREPT by Lovo cells in nude mice. (**L**). HDAC1 blocks the activity of CREPT in regulating the Wnt signaling pathway. Super-TOP-luciferase reporter (0.1 μg), pRL-TK (50 ng), HA–HDACs (0.1 μg) and Myc–CREPT (0.4 μg) were co-transfected into HEK293T cells. Luciferase activity is presented as a relative value based on the internal control (renilla signal). (**M**). HDAC1 inhibits the expression of CREPT-activated genes. The mRNA levels of CCND1, CLDN1, VEGFA, BMP4, PPARD, p21 and p27 were examined in DLD1 cell lines. (ns, not significantly, * *p* < 0.05, ** *p* < 0.01, *** *p* < 0.001).

Studies showed that CREPT could promote tumorigenesis but HDAC1 could promote and inhibit tumor growth [10,27,28,37,38,39,40,41,42]; we questioned whether HDAC1 and CREPT synergistically regulate tumor growth. To this end, a cell proliferation assay was performed in the colon cancer cell line DLD1 (Figure 2A,B). The results demonstrated that the ectopic expression of CREPT enhanced cell proliferation (Figure 2B, red triangle vs. black circle), but the ectopic expression of HDAC1 had no significant effect (Figure 2B, blue square vs. black circle). To our surprise, when HDAC1 was ectopically expressed in CREPT-overexpression cells, the cell proliferation was reduced (Figure 2B, orange reversed triangle vs. red triangle). Consistently, a colony formation experiment showed similar results (Figure 2C,D). All these results implied that HDAC1 might repress the activity of CREPT at tumor cell proliferation and colony formation. To confirm the role of endogenous HDAC1 on CREPT activity, we depleted CREPT and/or knocked down HDAC1 in the colon cancer cell line SW480 (Figure 2E,F). The data showed that the depletion of CREPT dramatically decreased cell proliferation (Figure 2F, red reversed triangle vs. black circle), but knocking down HDAC1 failed to affect cell growth (Figure 2F, blue square/triangle vs. black circle); however, knocking down HDAC1 based on the depletion of CREPT significantly recovered the cell proliferation (Figure 2F, orange circle/rhomb vs. red reversed triangle). A colony formation experiment showed similar results for the depletion of CREPT and HDAC1 (Figure 2G,H). To examine whether HDAC1 could inhibit CREPT-induced tumor formation, Lovo cells were subcutaneously injected into both sides of nude mice. The mice were sacrificed for analyses of tumor formation after 4 weeks. The results demonstrated that the tumors became larger in the mice inoculated with the CREPT overexpression cells than those in mock cells (Figure 2I–K). However, when HDAC1 was ectopically expressed in CREPT overexpression cells, the volume and mass of tumors were significantly reduced (Figure 2I–K). In summary, these data confirmed that HDAC1 counterpoised the activity of CREPT during tumorigenesis.

Based on previous studies that showed that CREPT promotes the Wnt signaling pathway in tumor cells [31,43] and other studies that showed that HDACs repress the Wnt target gene expression [44], we speculated whether HDAC1 could suppress CREPT-activated Wnt signaling. A luciferase reporter assay was used, and the results demonstrated that overexpressed CREPT significantly increased the luciferase activity upon Wnt stimulation (Figure 2L, Columns 3 vs. 2), but the over-expression of HDAC1 completely blocked the effect of CREPT (Figure 2L, Columns 7 vs. 3). Interestingly, the over-expression of HDAC2 or 3, the class I HDAC family members, showed no effect on CREPT-activated luciferase activity upon Wnt stimulation (Figure 2L, Columns 8 and 9 vs. 4 and 5). All these results suggest that HDAC1 specifically inhibits CREPT-mediated gene transcription.

To examine whether HDAC1 regulates the transcriptional activity of CREPT-mediated target genes in Wnt signaling, we performed a qRT-PCR analysis on DLD1 cell lines that stably over-expressed CREPT and/or HDAC1. The results showed that the mRNA levels of CCND1, CLDN1, VEGFA, PPARD and BMP4, the classical Wnt signal downstream genes, were up-regulated in CREPT overexpression cells but remained unchanged in HDAC1 overexpression cells (Figure 2M). However, the overexpression of HDAC1 reduced the mRNA levels of these genes in CREPT overexpression cells (Figure 2M, last two columns). These results suggest that HDAC1 repressed CREPT-regulated gene expression. Because these CREPT-upregulated genes are oncogenic-associated, we then further examined the expression of tumor suppressors. Interestingly, we observed that the overexpression of CREPT had no effect on the expression of p21 and p27, two well-known tumor suppressors, but the overexpression of HDAC1 significantly reduced their gene expression (Figure 2M). The overexpression of HDAC1 also repressed the p21 and p27 mRNAs upon CREPT-over-expression (Figure 2M, last two columns). These results suggest that HDAC1 represses tumor suppressors but not oncogenes while CREPT promotes oncogenes but not tumor suppressors (at least p21 and p27). Importantly, our data suggest that HDAC1 specifically represses CREPT-induced oncogene transcription.

### 3.3. HDAC1 Specifically Interacts with CREPT

**Figure 3:**
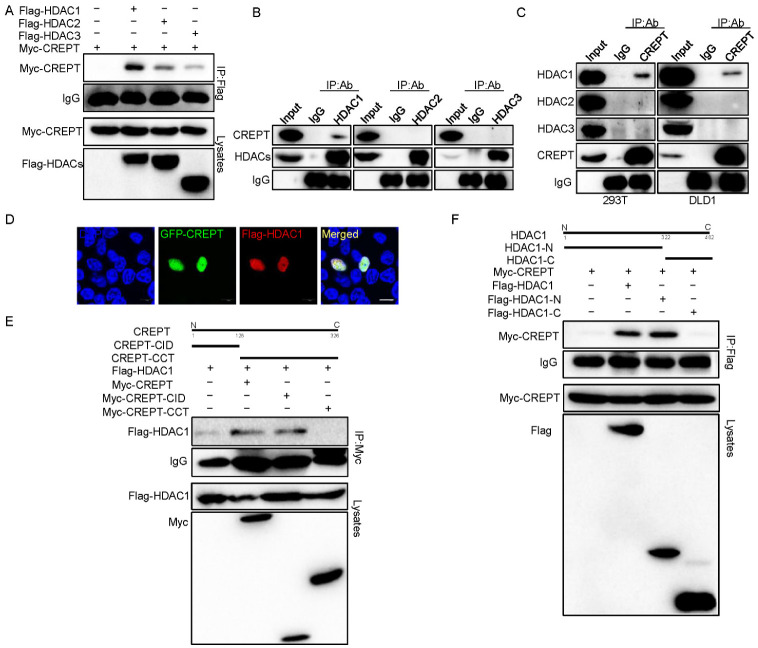
HDAC1 specifically interacts with CREPT. (**A**). Flag–HDAC1 interacts with Myc–CREPT. Myc–CREPT (3 μg) and Flag–HDAC1/2/3 (3 μg) were co-transfected into HEK293T cells for immunoprecipitation experiments. Antibodies used are indicated. (**B**,**C**). HDAC1 interacts with CREPT endogenously. Nuclear extracts were immunoprecipitated with an antibody against HDAC1/2/3 (**B**) or with an antibody against CREPT (**C**) in HEK293T and DLD1 cells. (**D**). HDAC1 and CREPT are co-localized in the nucleus. HEK293T cells expressing GFP-CREPT and Flag–HDAC1 were scanned by a confocal microscope after staining. Scale bar, 10 um. HDAC1 interacts with the CID domain of CREPT in HEK293T. Anti-Myc and anti-Flag antibodies were used. (**F**). The N-terminus domain of HDAC1 interacts with CREPT in HEK293T cells. Immunoprecipitation was performed (**E**).

To explore the molecular mechanism of how HDAC1 regulates the activity of CREPT, we determined to examine whether HDAC1 and CREPT interact. For this purpose, we performed an immunoprecipitation experiment using HEK293T cells, which express HA–HDAC1 and Myc–CREPT. The results showed that an antibody against HA strongly precipitated the Myc–CREPT protein (Figure 3A), suggesting that Myc–CREPT interacted with HA–HDAC1. To verify the interaction between CREPT and HDAC1 endogenously, we used antibodies against HDACs to perform the immunoprecipitation experiment. The results demonstrated that an antibody against HDAC1 precipitated CREPT, but HDAC2 and 3 failed (Figure 3B). Reciprocally, an antibody against CREPT precipitated HDAC1, but not HDAC2 and 3, in both HEK293T and DLD1 cells (Figure 3C). All these results suggest that endogenous HDAC1 specifically interacts with CREPT under physiological conditions.

To address whether CREPT and HDAC1 interact in intact cells, we performed an immunostaining experiment. The results showed that CREPT and HDAC1 co-localized in the nucleus (Figure 3D), suggesting that both proteins interact spatially. To map the interacting domains between HDAC1 and CREPT, we performed immunoprecipitation experiments using different domains of CREPT (CID and CCT domains [45]) and HDAC1 (N and C domains [13]). The results indicated that Myc–CREPT–CID precipitated Flag–HDAC1 but Myc–CREPT–CCT failed (Figure 3E), suggesting that the CID domain contributes to the CREPT and HDAC1 interaction. On the other hand, the immunoprecipitation results showed that the Flag–HDAC1-N domain precipitated Myc–CREPT but the Flag–HDAC1-C domain failed (Figure 3F), suggesting that the N domain is critical for the HDAC1 to interact with CREPT. These results firmly identified that HDAC1 specifically interacts with CREPT via the CID domain and the N domain.

### 3.4. HDAC1 and CREPT Exclusively Interact with TCF4 and β-Catenin

**Figure 4:**
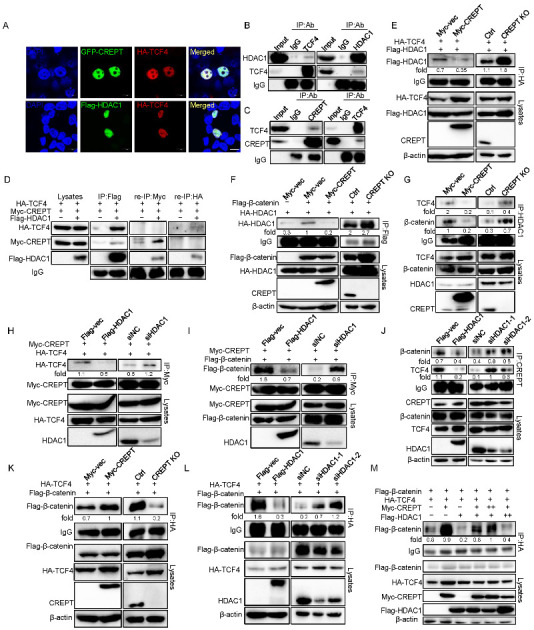
HDAC1 and CREPT exclusively interact with TCF4 and β-catenin. (**A**). HDAC1 and CREPT co-localize with TCF4 in the nucleus. HEK293T cells expressing HA–TCF4 together with GFP–CREPT or Flag–HDAC1 were scanned by confocal microscope. Scale bar, 10 um. (**B**). The interaction of HDAC1 with TCF4 is shown. Nuclear extracts from HEK293T cells were used. (**C**). CREPT associates with TCF4. Immunoprecipitation was performed with indicated antibodies. (**D**). TCF4 interacts with CREPT and HDAC1 exclusively. HEK293T cells were co-transfected with Flag–HDAC1 (10 μg), HA–TCF4 (6 μg) and Myc–CREPT (4 μg) for IP experiments using an antibody against Flag. Precipitants were used for re-IP experiments using an antibody against Myc or an antibody against HA. (**E**). CREPT impairs the TCF4–HDAC1 complex formation. HEK293T cells were co-transfected with Flag–HDAC1 (2 μg) and HA–TCF4 (2 μg) under stable expression (Myc–CREPT) or deletion (CREPT KO) of CREPT for IP experiments using an antibody against HA. (**F**). CREPT impedes the β-catenin–HDAC1 complex formation. HEK293T cells were co-transfected with HA–HDAC1 (2 μg) and Flag–β-catenin (2 μg) under stable expression (Myc–CREPT) or deletion (CREPT KO) of CREPT for IP experiments using an antibody against Flag. (**G**). CREPT represses the interaction between HDAC1 and β-catenin–TCF4 complex endogenously. HEK293T cells under stable expression (Myc–CREPT) or deletion (CREPT KO) of CREPT were collected for IP experiments using an antibody against HDAC1. (**H**,**I**). HDAC1 suppresses the CREPT–TCF4 complex (**H**) and CREPT–β-catenin complex (**I**) formation. HEK293T cells were co-transfected with Myc–CREPT (2 μg) and HA–TCF4 (2 μg) (**H**) or Flag–β-catenin (2 μg) (**I**) under overexpression (Flag–HDAC1) or deletion (siHDAC1) of HDAC1 for IP experiments using an antibody against Myc. (**J**). HDAC1 represses the interaction between CREPT and β-catenin–TCF4 complex endogenously. HEK293T cells under stable expression (Flag–HDAC1) or deletion (siHDAC1-1/2) of HDAC1 were collected for IP experiments using an antibody against CREPT. (**K**). CREPT facilitates the β-catenin–TCF4 complex formation. Cells were co-transfected with Flag–β-catenin (3 μg) and HA–TCF4 (3 μg) under stable expression (Myc–CREPT) or deletion (CREPT KO) of CREPT for IP experiments using an antibody against HA. The Myc–CREPT in the lysates was detected with anti-Myc antibody in the left panels of (**E**,**F**,**K**). The endogenous CREPT was detected with anti-CREPT antibody in the right panels of (**E**,**F**,**K**,**L**). HDAC1 impairs the β-catenin–TCF4 complex formation. HEK293T cells were co-transfected with Flag–β-catenin (3 μg) and HA–TCF4 (3 μg) under stable expression (Flag–HDAC1) or deletion (siHDAC1-1/2) of HDAC1 for IP experiments using an antibody against HA. The Flag–HDAC1 in the lysates was detected with anti-Flag antibody in the left panels of (**H**,**I**,**L**). The endogenous HDAC1 was detected with anti- HDAC1 antibody in the right panels of (**H**,**I**,**L**). (**M**). CREPT and HDAC1 competitively influence the interaction of β-catenin and TCF4. Flag–β-catenin (2 μg), HA–TCF4 (2 μg), Myc–CREPT (2 μg) (6th column) or (4 μg) (5th column), Flag–HDAC1 (2 μg) (5th column) or (4 μg) (6th column) were co-transfected in cells for IP experiment by using an antibody against HA.

Based on our previous studies that CREPT is associated with the transcription factor TCF4 in the nucleus to activate Wnt signaling [31], as well as other studies that show that HDAC1 binds to TCF4 to repress Wnt signaling activation [46], we speculated whether HDAC1 and CREPT could co-regulate Wnt signaling by TCF4 in mammalian cells. So, we explored the interactions among CREPT, HDAC1, and TCF4. An immunofluorescence experiment demonstrated that TCF4 co-localized with both CREPT and HDAC1 in the nucleus (Figure 4A), suggesting that CREPT and HDAC1 might interact with TCF4. Indeed, an immunoprecipitation experiment verified that both HDAC1 (Figure 4B) and CREPT (Figure 4C) interacted with TCF4 endogenously. To reveal whether CREPT, HDAC1, and TCF4 form a complex, we performed a re-immunoprecipitation experiment. The result showed that an antibody against Myc (for Myc–CREPT) precipitated only the Flag–HDAC1 protein, but not HA–TCF4, in the complex which was elucidated by an anti-Flag antibody (Figure 4D). Reciprocally, an antibody against HA (for HA–TCF4) only precipitated Flag–HDAC1 but not Myc–CREPT in the complex precipitated by the anti-Flag antibody (Figure 4D, right panel). These results suggest that CREPT and HDAC1 exclusively interact with TCF4, although CREPT and HDAC1 also interact.

The exclusive interaction of CREPT and HDAC1 with TCF4 implies that CREPT might interrupt the interaction of HDAC1 and TCF4. To reveal how CREPT regulates the interaction of HDAC1 and TCF4, we generated different cell lines where CREPT was stably overexpressed or depleted in HEK293T cells. An immunoprecipitation experiment demonstrated that the interaction between Flag–HDAC1 and HA–TCF4 was downregulated in CREPT overexpression cells, but increased in CREPT-depleted cells (Figure 4E). Furthermore, we observed that the interaction of Flag–β-catenin and HA–HDAC1 was repressed when CREPT was overexpressed but was enhanced when CREPT was depleted (Figure 4F). These results suggest that CREPT is able to interrupt the association of HDAC1 and TCF4 or β-catenin. This was further confirmed using endogenous proteins (Figure 4G).

On the other hand, we speculated that HDAC1 may also affect the binding of CREPT with TCF4/β-catenin. Immunoprecipitation experiments showed that the interaction of Myc–CREPT and HA–TCF4 was reduced in the presence of Flag–HDAC1 but was enhanced in the HDAC1-depleted cells (Figure 4H). Consistently, the overexpression of Flag–HDAC1 reduced, but the depletion of HDAC1 promoted the interaction of Myc–CREPT with Flag–β-catenin (Figure 4I). This was further confirmed using endogenous proteins (Figure 4J).These results suggest that HDAC1 blocks the interaction of CREPT with TCF4 or β-catenin.

To address whether CREPT and HDAC1 regulate the β-catenin/TCF4 complex, we performed a series of IP experiments. The results revealed that the complex formation of Flag–β-catenin and HA–TCF4 was enhanced in Myc–CREPT overexpression cells, but decreased in CREPT-depleted cells (Figure 4K). Conversely, the association between Flag–β-catenin and HA–TCF4 was reduced in Flag–HDAC1 overexpression cells but increased in HDAC1 depletion cells (Figure 4L). Interestingly, increasing Myc–CREPT expression counteracted the effect of overexpressed Flag–HDAC1 and vice versa (Figure 4M). Taken together, all these results indicate that CREPT promotes but HDAC1 represses the formation of the β-catenin-TCF4 complex. Of note, the interaction of CREPT and HDAC1 provided a base for their interplay on the β-catenin–TCF4 complex formation.

### 3.5. CREPT Blocks the Occupancy of HDAC1 at the TBS Region of Target Genes

**Figure 5:**
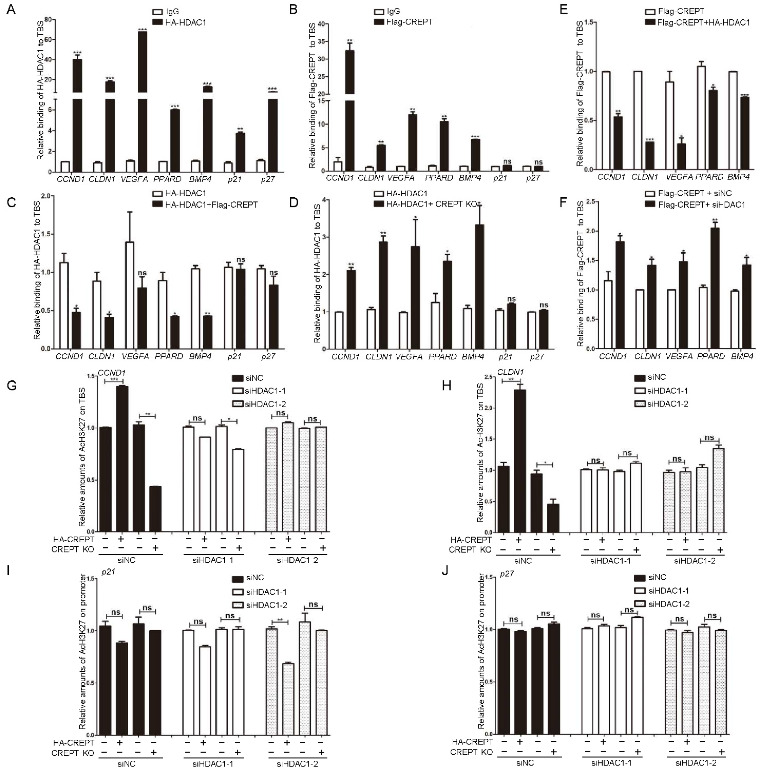
CREPT blocks the occupancy of HDAC1 at the TBS region of target genes. (**A**,**B**). HDAC1 or CREPT occupies the TCF4 binding sequence (TBS) of Wnt target oncogenes. DLD1 cells overexpressing HDAC1 (**A**) or CREPT (**B**) were collected for the ChIP-qPCR assay. (**C**). CREPT represses the binding of HDAC1 on the TBS region of Wnt target oncogenes. DLD1 cells under stable expression of HA–HDAC1 and Flag–CREPT were collected for ChIP-qPCR assay. (**D**). Deletion of CREPT facilitates the occupancy of HDAC1 on the TBS region. DLD1 cells under deletion of CREPT were cultured in the presence of over-expressed HA–HDAC1 and harvested for ChIP-qPCR assay using an antibody against HA. (**E**). HDAC1 impairs the binding of CREPT on the TBS region of Wnt target oncogenes. DLD1 cells under stable expression of HA–HDAC1 and Flag–CREPT were collected for ChIP-qPCR assay. (**F**). Deletion of HDAC1 promotes the occupancy of CREPT at the TBS region. DLD1 cells under deletion of HDAC1 were cultured in the presence of over-expressed Flag–CREPT and harvested for ChIP-qPCR assay using an antibody against Flag. The occupancy abundance was revealed by the precipitated DNA fragments on the TBS regions using qPCR. Wnt target oncogenes CCND1, CLDN1, VEGFA, PPARD, and BMP4 were examined. Tumor suppressor genes p21 and p27 were used as a control. (**G**,**H**). CREPT regulates the histone acetylation at H3K27 in the promoters of CCND1 (**G**) or CLDN1 (**H**) depending on HDAC1. (**I**,**J**). CREPT failed to influence the acetylation levels of tumor suppressor genes p21 (**I**) or p27 (**J**). Cells under stable expression (HA–CREPT) or deletion (CREPT KO) of CREPT were transfected with siRNAs against HDAC1 (si-HDAC1-1, siHDAC1-2). ChIP-qPCR was performed using an antibody against AcH3K27. (ns, not significantly, * *p* < 0.05, ** *p* < 0.01, *** *p* < 0.001).

To address how CREPT and HDAC1 interplay to influence the activity of the β-catenin–TCF4 complex, we identified whether CREPT and HDAC1 occupy the promoter region that TCF4 binds. For this purpose, we performed chromatin immunoprecipitation (ChIP) qPCR experiment to amplify the TBS (TCF4 binding sequence) regions of Wnt target genes, including *CCND1*, *CLDN1*, *VEGFA*, *PPARD* and *BMP4*. The results revealed that both HDAC1 (Figure 5A) and CREPT (Figure 5B) occupied the TBS region in DLD1 cells significantly. On the other hand, as a control, we examined the occupancy of CREPT and HDAC1 at the promoters of tumor suppressor genes such as p21 and p27, which are not regulated by TCF4. The results showed that CREPT failed to occupy these promoters (Figure 5B, last two sets of columns), whereas HDAC1 showed a strong occupancy (Figure 5A, last two sets of columns). All the results suggest that both HDAC1 and CREPT are involved in regulating the expression of oncogenes, but only HDAC1 participates in the regulation of tumor suppressors while CREPT appears to not, at least in p21 and p27. These results are consistent with the results of gene expression as examined by RT-qPCR (see Figure 2M).

The strong occupancy of CREPT and HDAC1, as well as their interaction, reminded us to examine whether their occupancy at the promoters is concurrent. A ChIP-qPCR analysis indicated that the overexpression of CREPT significantly reduced (Figure 5C), but depletion of CREPT increased (Figure 5D), the binding of HDAC1 at the TBS regions compared with that in control cells. However, we observed that CREPT failed to influence the occupancy of HDAC1 at the tumor suppressor promoters (Figure 5C,D, p21 and p27).

Reciprocally, we observed that the overexpression of HDAC1 impaired (Figure 5E), but the depletion of HDAC1 increased (Figure 5F), the occupancy of CREPT at the promoter regions. These results suggested that CREPT represses the binding of HDAC1 at the promoter regions of Wnt downstream oncogenes but not tumor suppressors.

To address whether CREPT regulates the HDAC1 activity on histones, we detected the acetylated level of histone 3 (Ac-H3K27). The ChIP-qPCR results revealed that the level of Ac-H3K27 at the promoter region of the *CCND1* gene was strongly enhanced when CREPT was overexpressed (Figure 5G, compared second with first column). Conversely, the deletion of CREPT decreased Ac-H3K27 level significantly (Figure 5G, compared fourth to third column). Similar results were observed at the promoters of *CLDN1* (Figure 5H). These results suggested that CREPT promotes the acetylation level of H3K27 at the promoters of Wnt downstream oncogenes. However, CREPT failed to influence the acetylation levels of tumor suppressors such as p21 and p27 (Figure 5I,J), which is consistent with the fact that CREPT failed to affect the expression of p21 and p27 (see Figure 2M). Intriguingly, we observed that the overexpression or depletion of CREPT failed to affect the Ac-H3K27 level under the HDAC1 depletion condition (Figure 5G,H). This result suggests that CREPT competes with HDAC1 and maintains the acetylation of H3K27.

### 3.6. Wnt3a Influences the Binding of HDAC1/CREPT to the TBS Region

**Figure 6:**
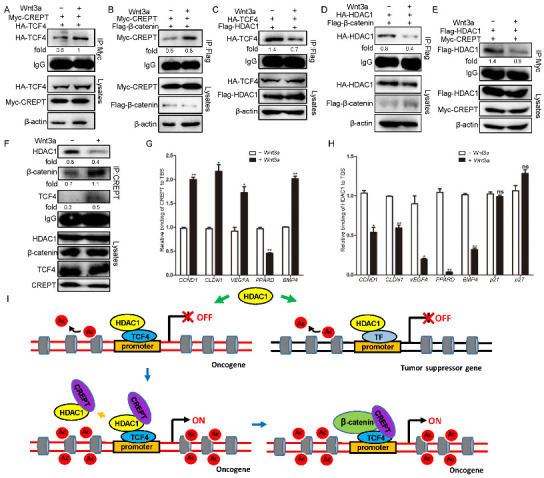
Wnt3a induces CREPT but reduces HDAC1 binding TBS. (**A**,**B**). Wnt3a increases the interaction between CREPT and TCF4 (**A**)-β-catenin (**B**) complex. Myc–CREPT (3 μg), HA–TCF4 (3 μg) (**A**) or Flag–β-catenin (3 μg) (**B**) were co-transfected in HEK293T cells. Then, Wnt3a was added for 20 h. (**C**,**D**). Wnt3a represses the interaction between HDAC1 and TCF4 (**C**)/β-catenin (**D**) complex. Flag–HDAC1 (3 μg), HA–TCF4 (3 μg) (**C**) or Flag–β-catenin (3 μg) (**D**) were co-transfected cells with or without Wnt3a for 20 h. (**E**). Wnt3a decreases the interaction between CREPT and HDAC1. HEK293T cells were transfected with Myc–CREPT (3 μg) and Flag–HDAC1 (3 μg) in the presence or absence of Wnt3a. (**F**). Wnt3a increases the interaction of CREPT with TCF4/β-catenin but reduces the CREPT–HDAC1 interaction. (**G**). Wnt3a increases the binding of CREPT on TBS regions. (**H**). Wnt3a reduces the occupancy of HDAC1 on the TBS regions. HEK293T cells were harvested with or without Wnt3a for 24 h. Then cells were collected for a ChIP assay using indicated antibodies. The ChIPed DNA was examined by qPCR. (**I**). A model demonstrating the competition of CREPT with HDAC1 in regulating gene expression during tumorigenesis. In tumor cells, HDAC1 interacts with transcription factors and maintains histone proteins at the promoters of tumor suppressor genes (TSG) in a deacetylation state (right panel), leading to repressed TSG transcription. However, highly expressed CREPT is recruited to the promoter of Wnt target oncogenes. Then, CREPT interacts and dissociates HDAC1 from TCF4. The dissociation of HDAC1 recovers the promoter in an acetylated state. The subsequent binding of CREPT to TCF4/β-catenin initiates oncogene transcription and promotes tumorigenesis. (ns, not significantly, * *p* < 0.05, ** *p* < 0.01).

To explore whether Wnt signaling influences the interaction of CREPT or HDAC1 with the TCF4/β-catenin complex. Immunoprecipitation experiments demonstrated that the interaction of HA–TCF4 (Figure 6A) or Flag–β-catenin (Figure 6B) with Myc–CREPT was increased after Wnt3a treatment. On the contrary, the interaction of TCF4 (Figure 6C) or β-catenin (Figure 6D) with HDAC1 was impaired in the presence of Wnt signaling. Concomitantly, we observed that Wnt3a decreased the interaction of CREPT and HDAC1 (Figure 6E). Simultaneously, Wnt3a increased the interaction of CREPT with TCF4/β-catenin but reduced the endogenous CREPT-HDAC1 interaction (Figure 6F). Furthermore, ChIP-qPCR analyses indicated that the binding of CREPT to the promoters of several oncogenes (*CCND1*, *CLDN1*, *VEGFA* and *BMP4*, but not *PPARD*) were increased (Figure 6G), while the binding of HDAC1 to the promoters was decreased after Wnt3a treatment (Figure 6H). However, the Wnt3a treatment failed to influence the binding of HDAC1 at the tumor suppressors (at least p21 and p27) (Figure 6H, last two sets of columns). All the results suggest that Wnt signaling activates the binding of CREPT which then competes HDAC1 at the promoter regions of Wnt downstream oncogenes.

## 4. Discussion

HDACs, in particular HDAC1, have been widely reported to negatively regulate gene expression by the modification of lysine residues in histones via the deacetylation process [47,48]. Highly expressed HDAC1 maintains the chromatin in a closed status, leading to an inaccessible binding of transcription factors and other co-activators. Many studies have revealed that elevated HDAC1 represses tumor suppressor gene expression in tumors, and inhibition of HDAC1 resulted in the initiation of tumor suppressor expression [9,18,49]. However, one could argue why elevated HDAC1 fails to inhibit oncogene expression. Actually, the overexpression of HDAC1 was reported to inhibit oncogene expression in the absence of Wnt stimulation [50]. Therefore, whether a high level of HDAC1 represses tumor suppressor or oncogene expression is a question. In this study, we deciphered the mechanism of the paradoxical roles of HDAC1 in the regulation of tumor suppressors and oncogenes. Our results built up a model that HDAC1 is competed with by CREPT at the promoters of oncogenes, but remains at the promoters of tumor suppressor genes. Since CREPT mainly occupies the oncogene promoters but not the tumor suppressor promoters, we reasoned that its interaction with HDAC1 protects the oncogene promoters from being deacetylated, but has no effect on the HDAC1 occupancy of the tumor suppressors.

Our model is based on the exclusive interaction of CREPT and HDAC1 with TCF4 and β-catenin (Figure 6I). Our data showed that CREPT promotes Wnt/β-catenin signaling and tumorigenesis, but HDAC1 is able to counteract with CREPT and represses its downstream gene expression. We propose that the dissociation or the maintenance of HDAC1 at the promoters of oncogenes or tumor suppressor genes in cancer cells is dependent on the presence of CREPT. We observed that CREPT competes with HDAC1 to regulate the transcription of Wnt downstream genes by interfering with the β-catenin/TCF4 complex formation. This is consistent with the observations that CREPT recruits p300 to promote the p300-mediated acetylation of β-catenin, leading to increased β-catenin–TCF4 complex formation [32]. CREPT blocks the binding of HDAC1 at the promoter regions of Wnt target genes, thus making chromatin accessible for the binding of β-catenin and activating transcription. The role of CREPT on the HDAC1 dissociation at the promoters facilitates oncogene transcription. On the other hand, we found that CREPT fails to affect the occupancy of HDAC1 at the tumor suppressor promoters. All the results from clinic and cellular experiments were in consistence to support our model.

Previous studies reported that both HDAC1 and HDAC2 inhibit the transcription of the Wnt signaling pathway. However, in this research, we found that HA–HDAC2 interacted with Myc–CREPT, but endogenous HDAC2 failed to interact with CREPT in HEK293T cells (Figure 3A–C). Furthermore, our data identified that HDAC2 could not impact CREPT-mediated super-Top luciferase signaling after Wnt3a treatment (Figure 2L). We considered that HDAC2 might function independently of CREPT. For example, HDAC2 directly interacts with p15RS to suppress the Wnt signaling pathway [33]. CREPT and p15RS are homologous proteins but have opposite functions in cells. HDAC1 and HDAC2 may reciprocally recruit different factors to influence different gene transcription. Consistent with our hypothesis, several reports have demonstrated the differential roles of HDAC1 and HDAC2 [51]. For example, studies showed that HDAC2 predominates for oocyte development, but HDAC1 is crucial for preimplantation development [52]. Another study demonstrated that HDAC1 was upregulated but HDAC2 was downregulated in the centrolateral amygdala [53]. Nevertheless, our study confirmed that CREPT specifically interacts with HDAC1, but not HDAC2, to regulate the Wnt signaling pathway.

To date, the FDA has approved several HDAC inhibitors including Vorinostat, Romidepsin, Belinostat and Panobinostat for cancer therapy [9,54,55,56]. However, the clinical output is far from satisfactory in solid tumors. We speculate that the reasons could be due to the fact that the inhibition of HDAC has a limited role in recovering tumor suppressors but releases the activity of highly expressed oncoproteins such as CREPT. We imagine that the inhibition of the oncoprotein CREPT could be a powerful way to complement the cancer therapy by HDAC inhibitors.

## 5. Conclusions

Our studies explain the mechanism of the paradoxical roles of HDAC1 in the regulation of tumor suppressors and oncogenes. We report that elevated CREPT prevents HDAC1 from binding to the promoters of oncogenes but has no effect on the promoters of tumor suppressors during tumorigenesis. This work is critical for the cancer field at direction of therapy strategy and the development of new drugs.

## Data Availability

The data presented in this study are available in the article and Supplementary Materials.

## Supplementary Materials

The following supporting information can be downloaded at: https://www.mdpi.com/article/10.3390/cancers14194797/s1, Supplementary List S1: qRT-PCR primers; Supplemental Figure S1: The correlation of HDAC1 and CREPT in human cancers. A. CREPT expression is positively correlated to HDAC1 at the protein level in breast cancer cells. The endogenous protein levels of HDAC1 and CREPT in breast cancer cell lines (T47D, MCF7, MDA231) and epithelial cell (MCF10A) were shown. β-actin was used as a loading control. BC. The mRNA level of HDAC1 and CREPT in breast cancer cells compared with the normal cell. The mRNA levels were normalized to fold change relative to β-actin. D. HDAC1 and CREPT are highly correlated in TCGA (The Cancer Genome Atlas) database. BRCA represents breast invasive carcinoma, LUAD represents lung adenocarcinoma, PRAD represents prostate adenocarcinoma.
