# Supplementary material for: CREPT Disarms the Inhibitory Activity of HDAC1 on Oncogene Expression to Promote Tumorigenesis
Source: Cancers (Basel). 2022 Sep 30;14(19):4797. doi: 10.3390/cancers14194797 (PMC9562184; doi:10.3390/cancers14194797)
Supplement: Supplementary file 1 [file cancers-14-04797-s001.zip › cancers-1842508-supplementary/Supplementary Figure S1.pdf]

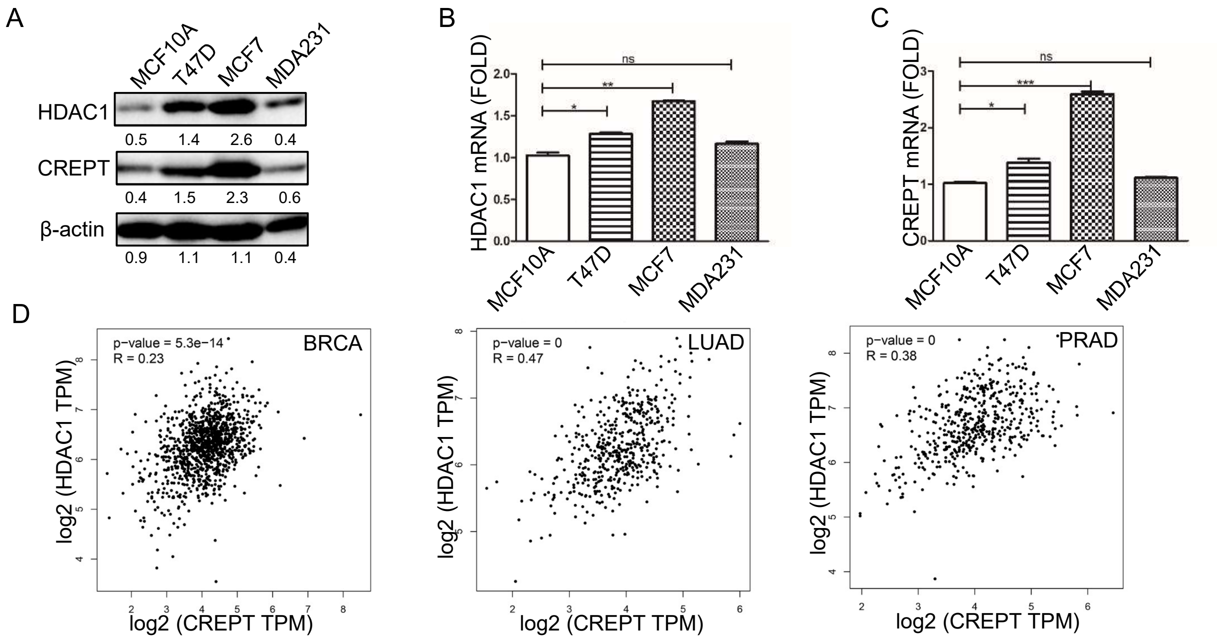

Supplementary Figure S1. The correlation of HDAC1 and CREPT in human cancers. A. CREPT expression is positively correlated to HDAC1 at the protein level in breast cancer cells. The endogenous protein levels of HDAC1 and CREPT in breast cancer cell lines (T47D, MCF7, MDA231) and epithelial cell (MCF10A) were shown. β-actin was used as a loading control. B-C. The mRNA level of HDAC1 and CREPT in breast cancer cells compared with the normal cell. The mRNA levels were normalized to fold change relative to β-actin. D. HDAC1 and CREPT are highly correlated in TCGA (The Cancer Genome Atlas) database. BRCA represents breast invasive carcinoma, LUAD represents lung adenocarcinoma, PRAD represents prostate adenocarcinoma.
