# Supplementary material for: CREPT Disarms the Inhibitory Activity of HDAC1 on Oncogene Expression to Promote Tumorigenesis
Source: Cancers (Basel). 2022 Sep 30;14(19):4797. doi: 10.3390/cancers14194797 (PMC9562184; doi:10.3390/cancers14194797)
Supplement: Supplementary file 1 [file cancers-14-04797-s001.zip › cancers-1842508-supplementary/Supplementary Figures original Western blots.pdf]

Figure S1A

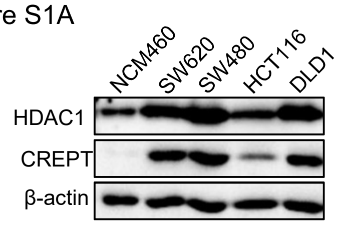

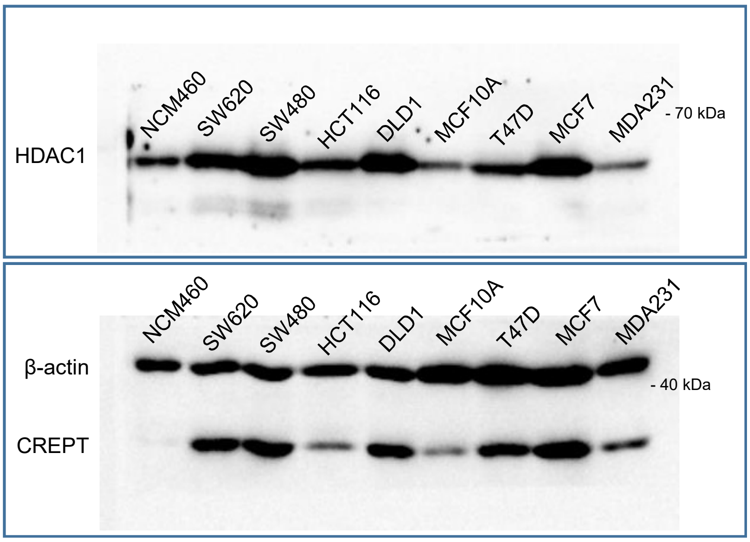

Supplementary Figure S1A

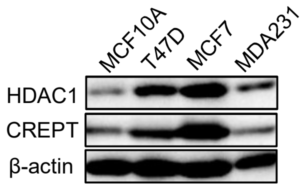

Figure S1D

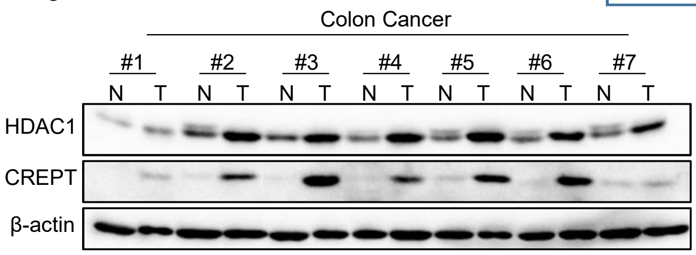

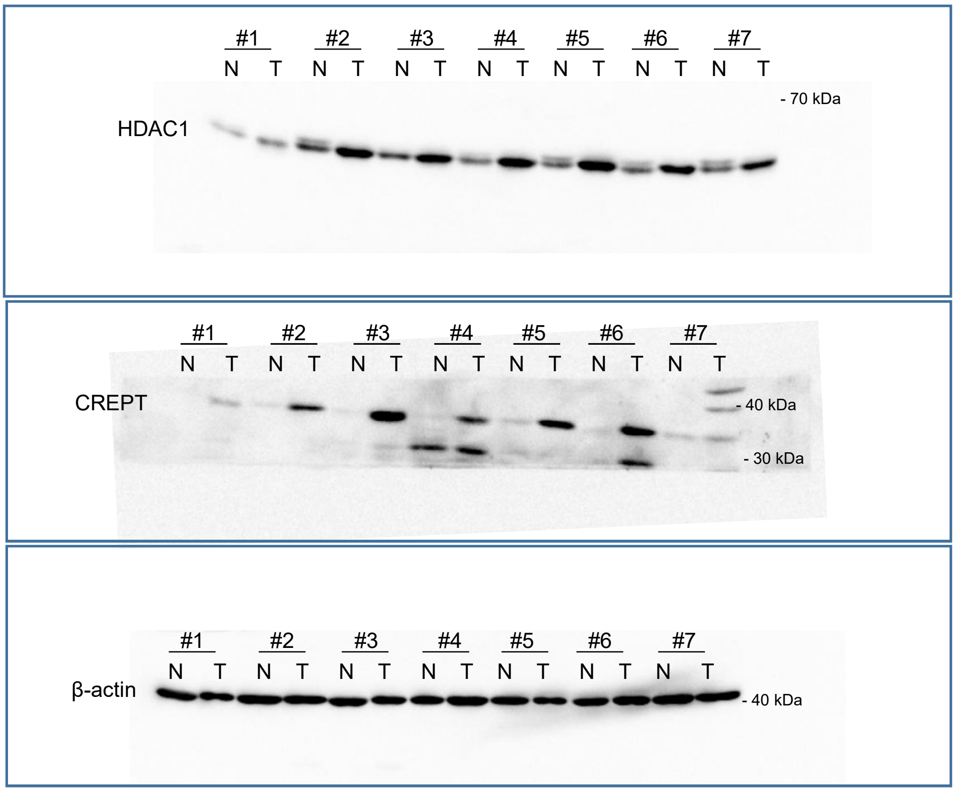

Figure S2A

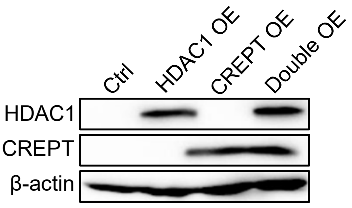

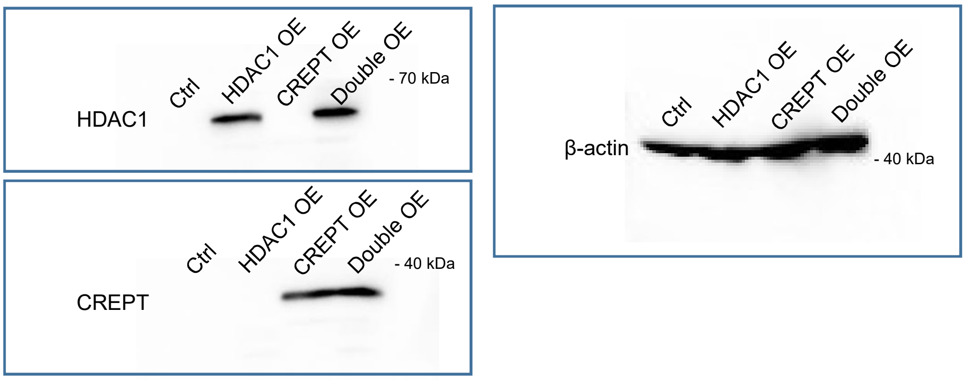

Figure S2E

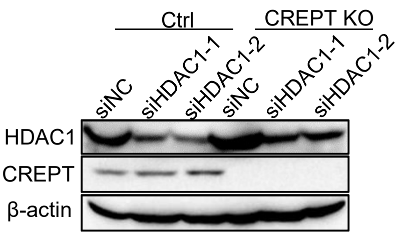

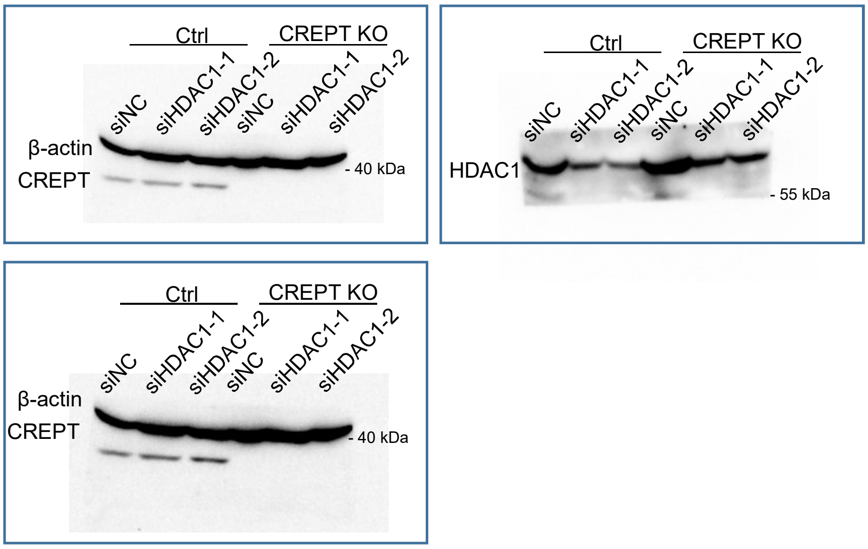

Figure S2L

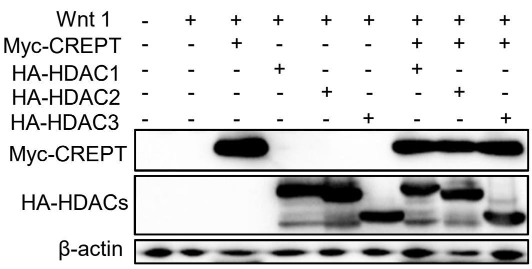

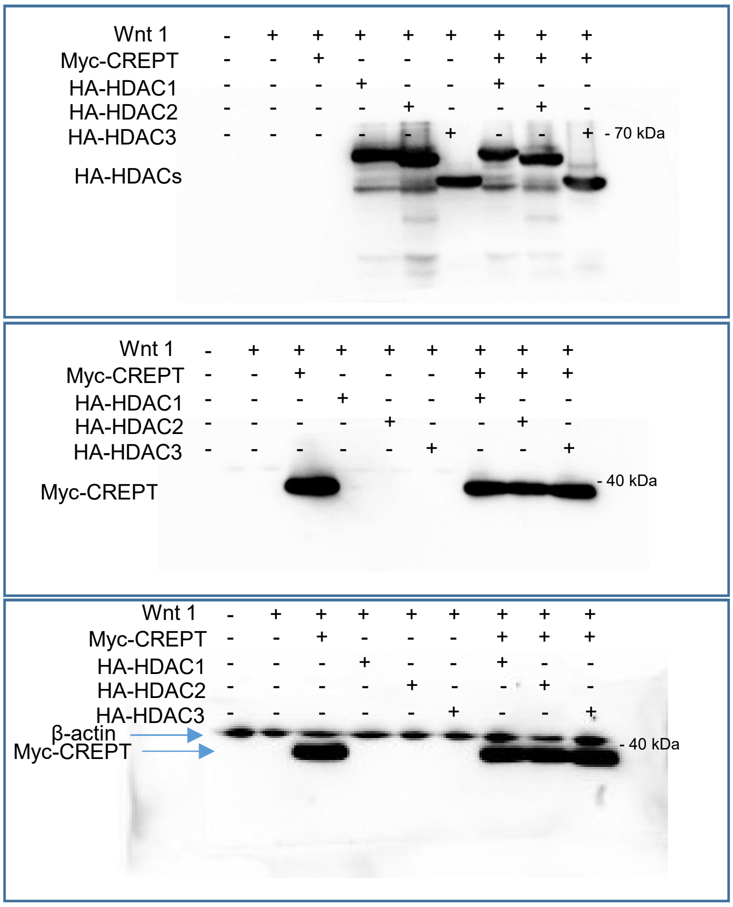

Figure S3A

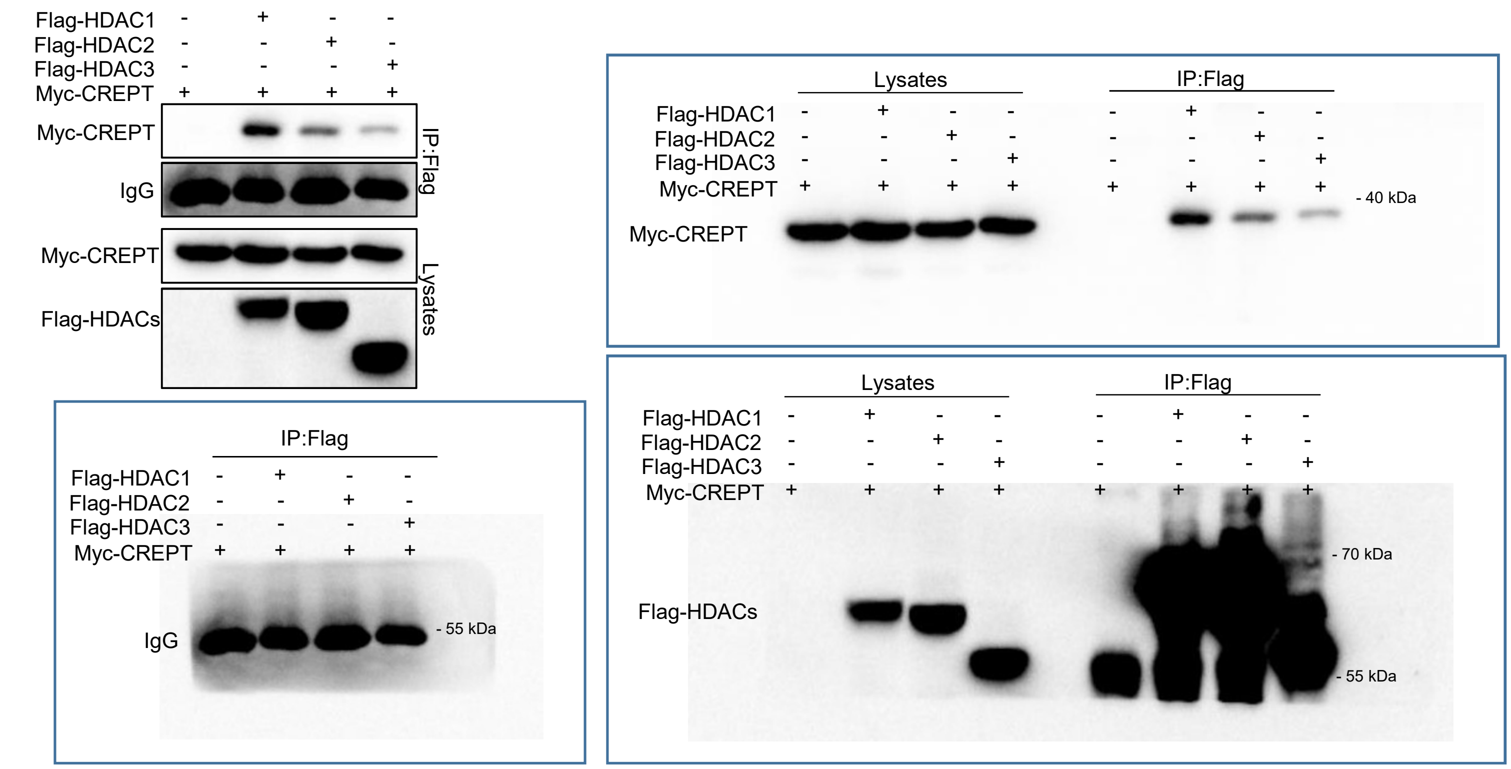

Figure S3B

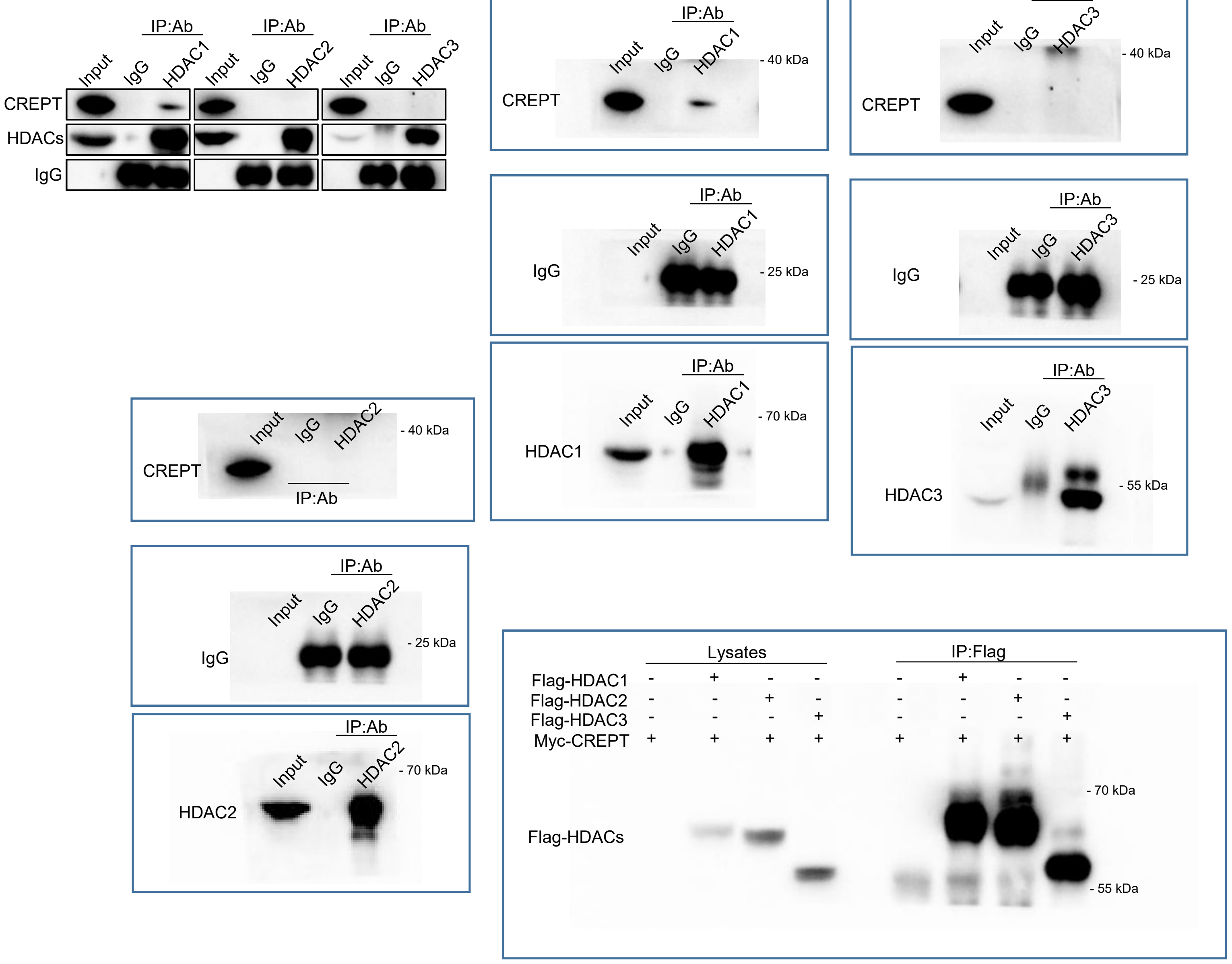

Figure S3C

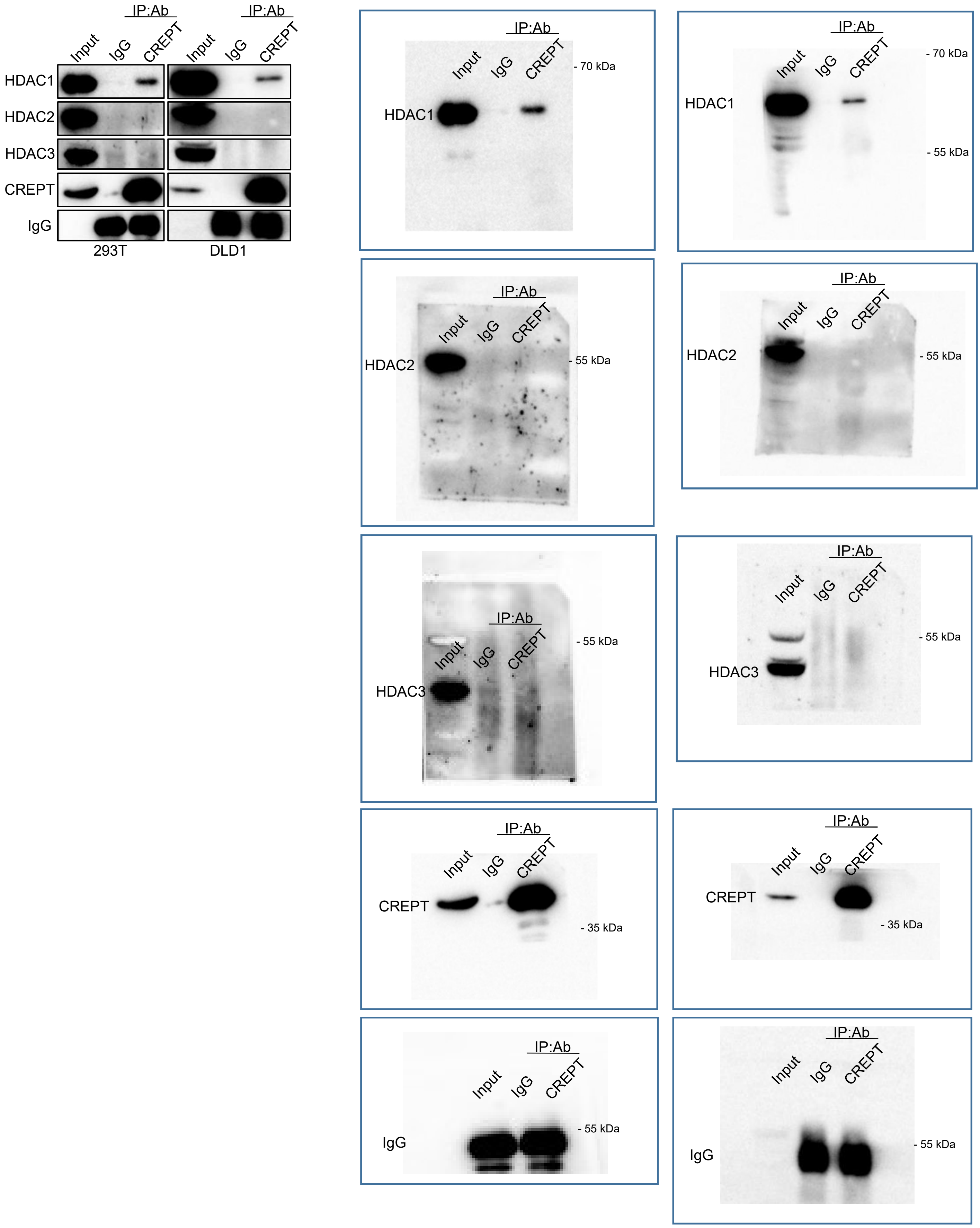

293T
DLD1
IP:Ab
Input
IgG
CREPT
HDAC1
HDAC2
HDAC3
CREPT
IgG
293T
DLD1
- 70 kDa
- 55 kDa
- 35 kDa

Figure S3E

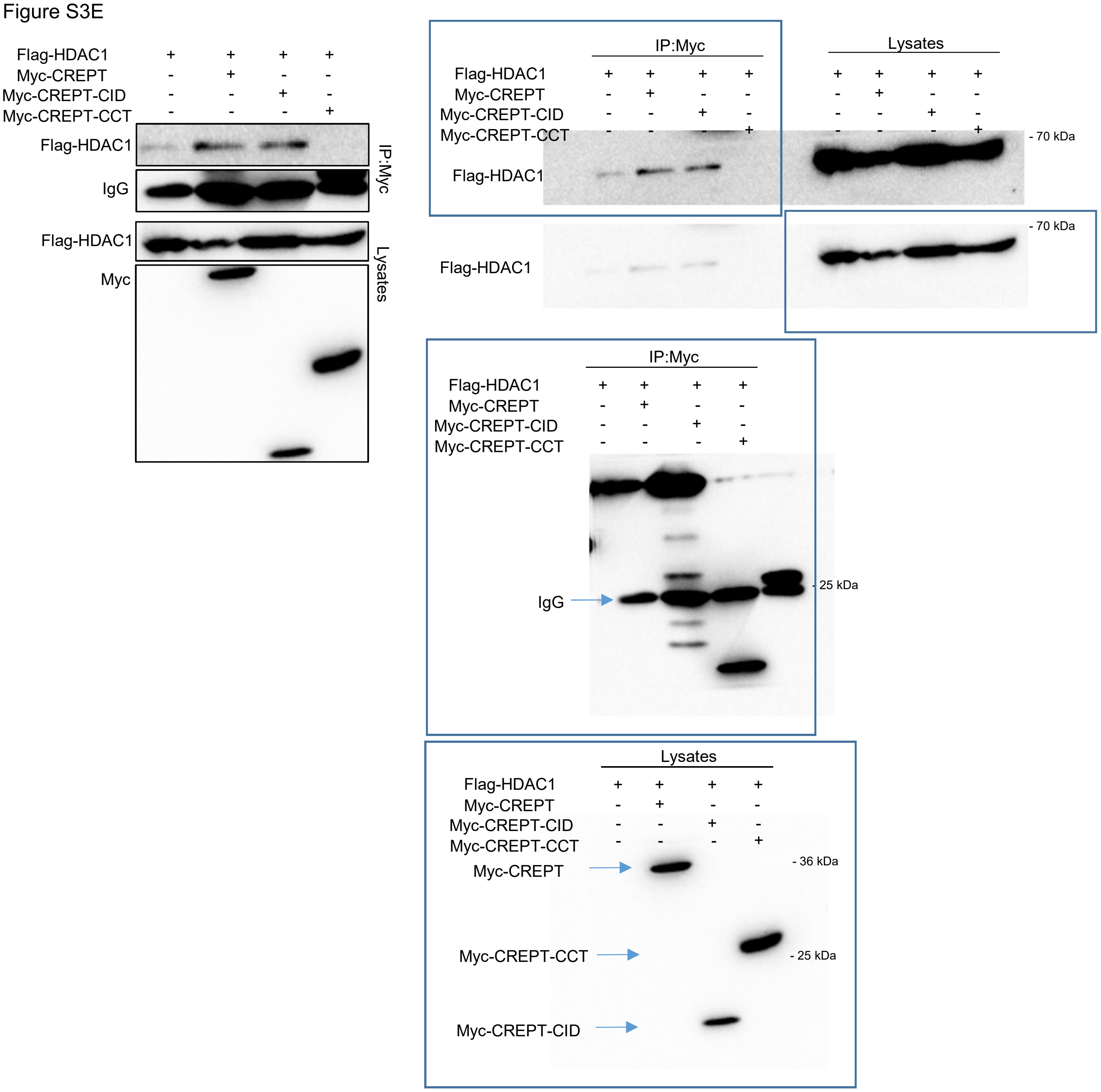

Flag-HDAC1
Myc-CREPT
Myc-CREPT-CID
Myc-CREPT-CCT
Flag-HDAC1
IgG
IP:Myc
Flag-HDAC1
Myc
Lysates
IP:Myc
Lysates
Flag-HDAC1
Myc-CREPT
Myc-CREPT-CID
Myc-CREPT-CCT
Flag-HDAC1
- 70 kDa
Flag-HDAC1
- 70 kDa
IP:Myc
Flag-HDAC1
Myc-CREPT
Myc-CREPT-CID
Myc-CREPT-CCT
IgG
- 25 kDa
Lysates
Flag-HDAC1
Myc-CREPT
Myc-CREPT-CID
Myc-CREPT-CCT
Myc-CREPT
- 36 kDa
Myc-CREPT-CCT
- 25 kDa
Myc-CREPT-CID

Figure S3F

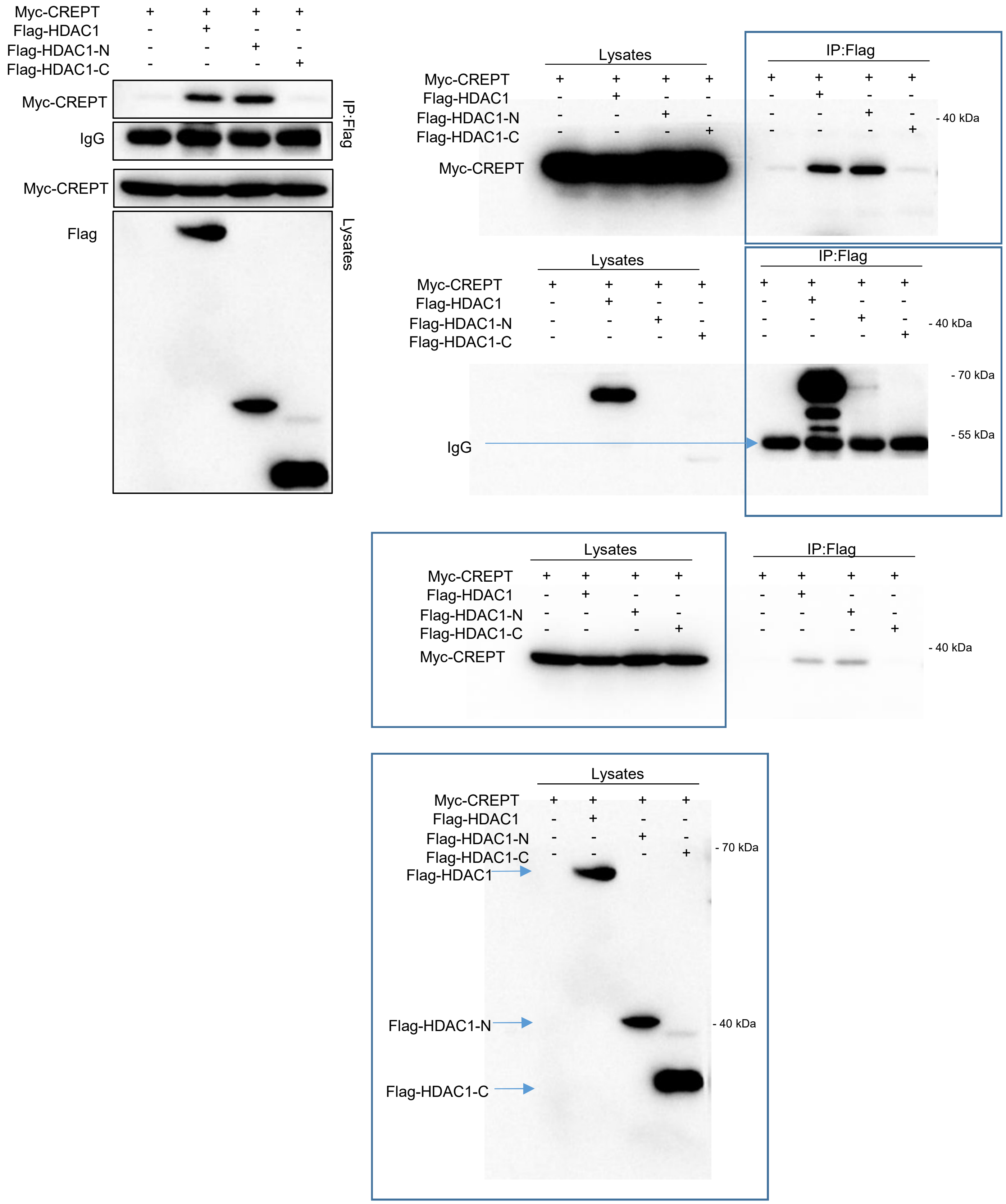

Myc-CREPT
Flag-HDAC1
Flag-HDAC1-N
Flag-HDAC1-C
Myc-CREPT
IgG
IP:Flag
Myc-CREPT
Flag
Lysates
Lysates
IP:Flag
Myc-CREPT
Flag-HDAC1
Flag-HDAC1-N
Flag-HDAC1-C
Myc-CREPT
- 40 kDa
Lysates
IP:Flag
Myc-CREPT
Flag-HDAC1
Flag-HDAC1-N
Flag-HDAC1-C
- 40 kDa
- 70 kDa
IgG
- 55 kDa
Lysates
IP:Flag
Myc-CREPT
Flag-HDAC1
Flag-HDAC1-N
Flag-HDAC1-C
Myc-CREPT
- 40 kDa
Lysates
Myc-CREPT
Flag-HDAC1
Flag-HDAC1-N
Flag-HDAC1-C
- 70 kDa
Flag-HDAC1
Flag-HDAC1-N
- 40 kDa
Flag-HDAC1-C

Figure S4B

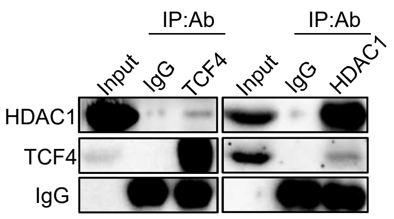

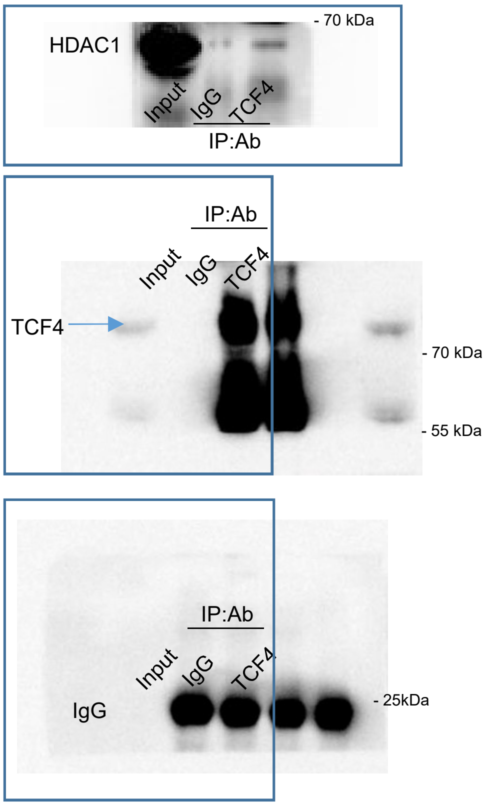

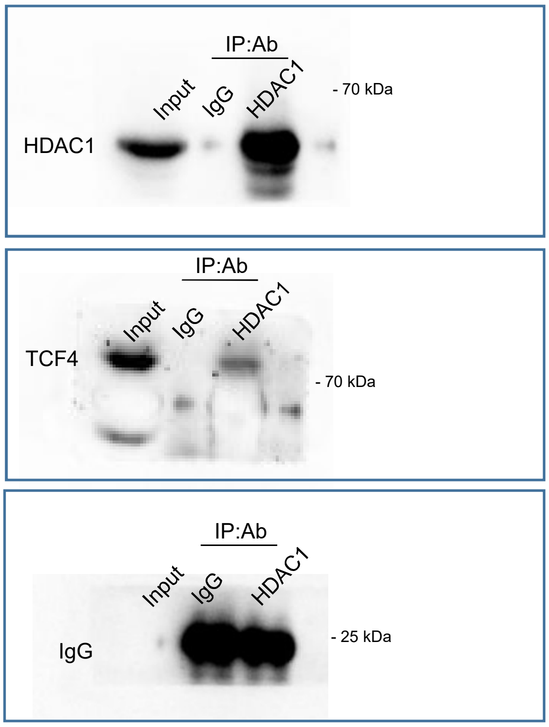

This samples were same with Fig.3B (IP:HDAC1)

The Molecular Wt. of TCF4 antibody:58,79kDa

Figure S4C

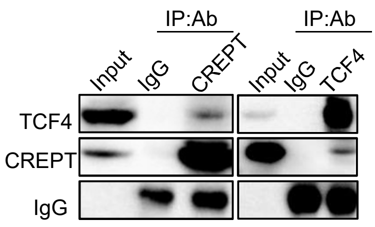

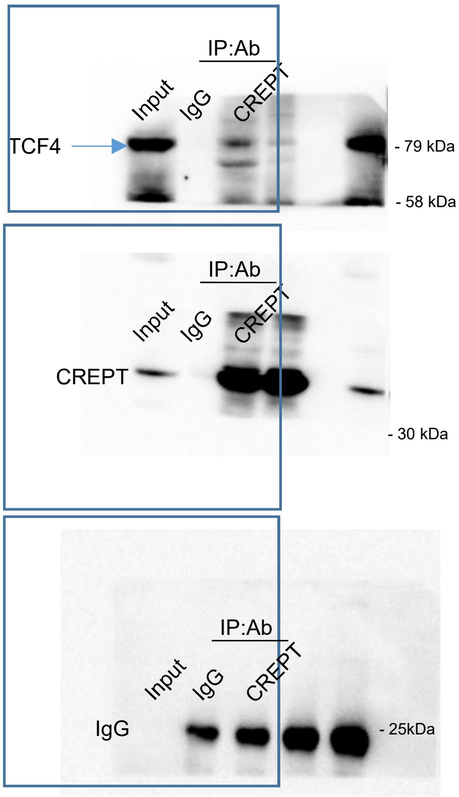

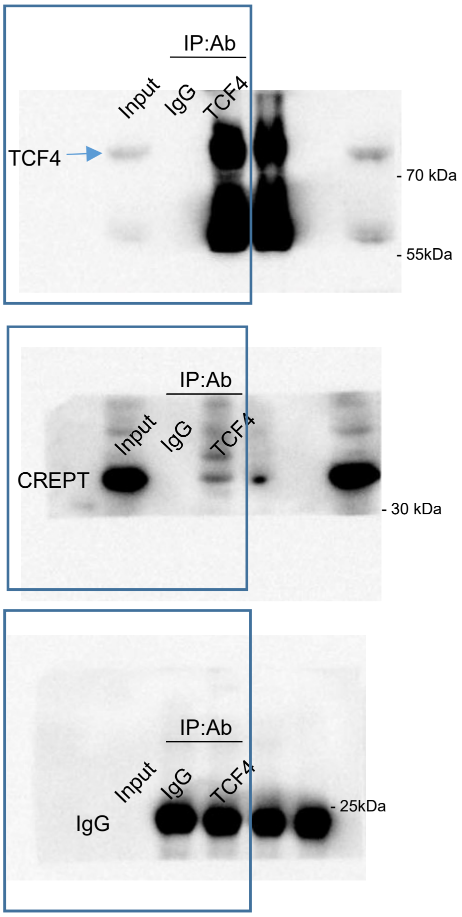

This samples were same with Fig.4B (IP:TCF4)

Figure S4D

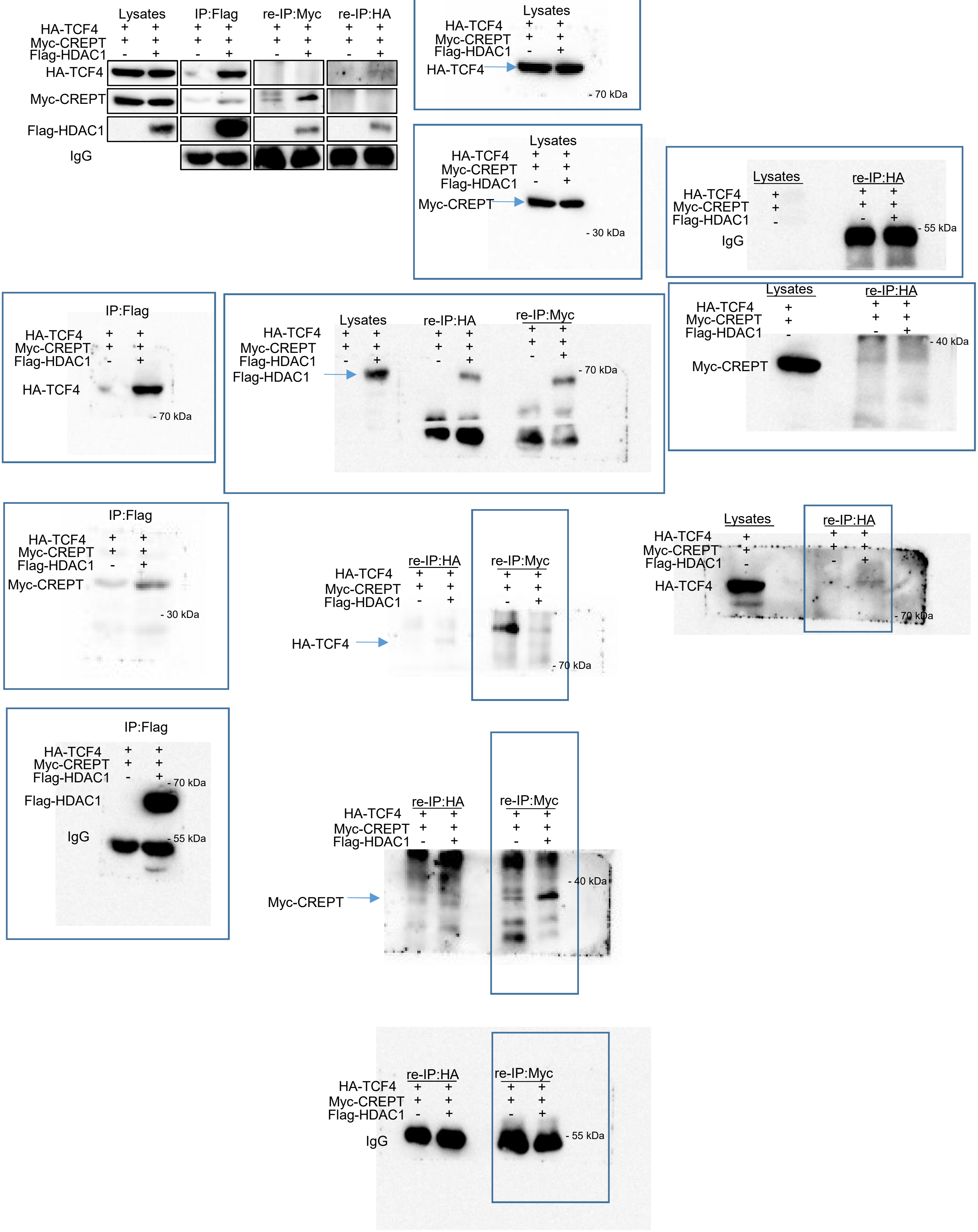
Lysates
IP:Flag
re-IP:Myc
re-IP:HA
HA-TCF4
Myc-CREPT
Flag-HDAC1
IgG
- 70 kDa
- 30 kDa
- 55 kDa
- 40 kDa

Figure S4E

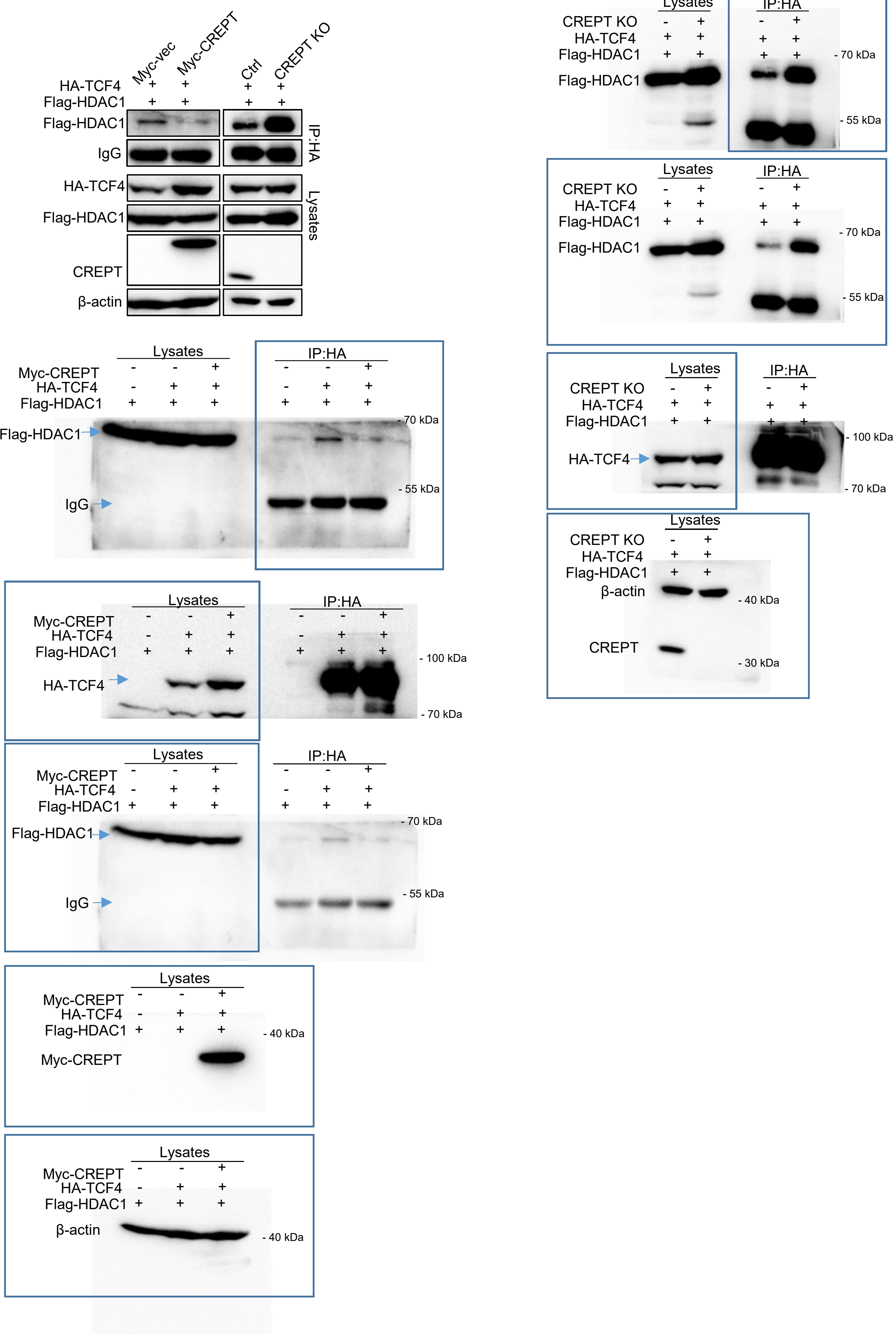
Myc-vec
Myc-CREPT
Ctrl
CREPT KO
HA-TCF4
Flag-HDAC1
Flag-HDAC1
IgG
HA-TCF4
Flag-HDAC1
CREPT
β-actin
IP:HA
Lysates
Lysates
IP:HA
Myc-CREPT
HA-TCF4
Flag-HDAC1
Flag-HDAC1
IgG
- 70 kDa
- 55 kDa
- 100 kDa
- 40 kDa
- 30 kDa
CREPT KO
HA-TCF4
Myc-CREPT
β-actin

Figure S4F

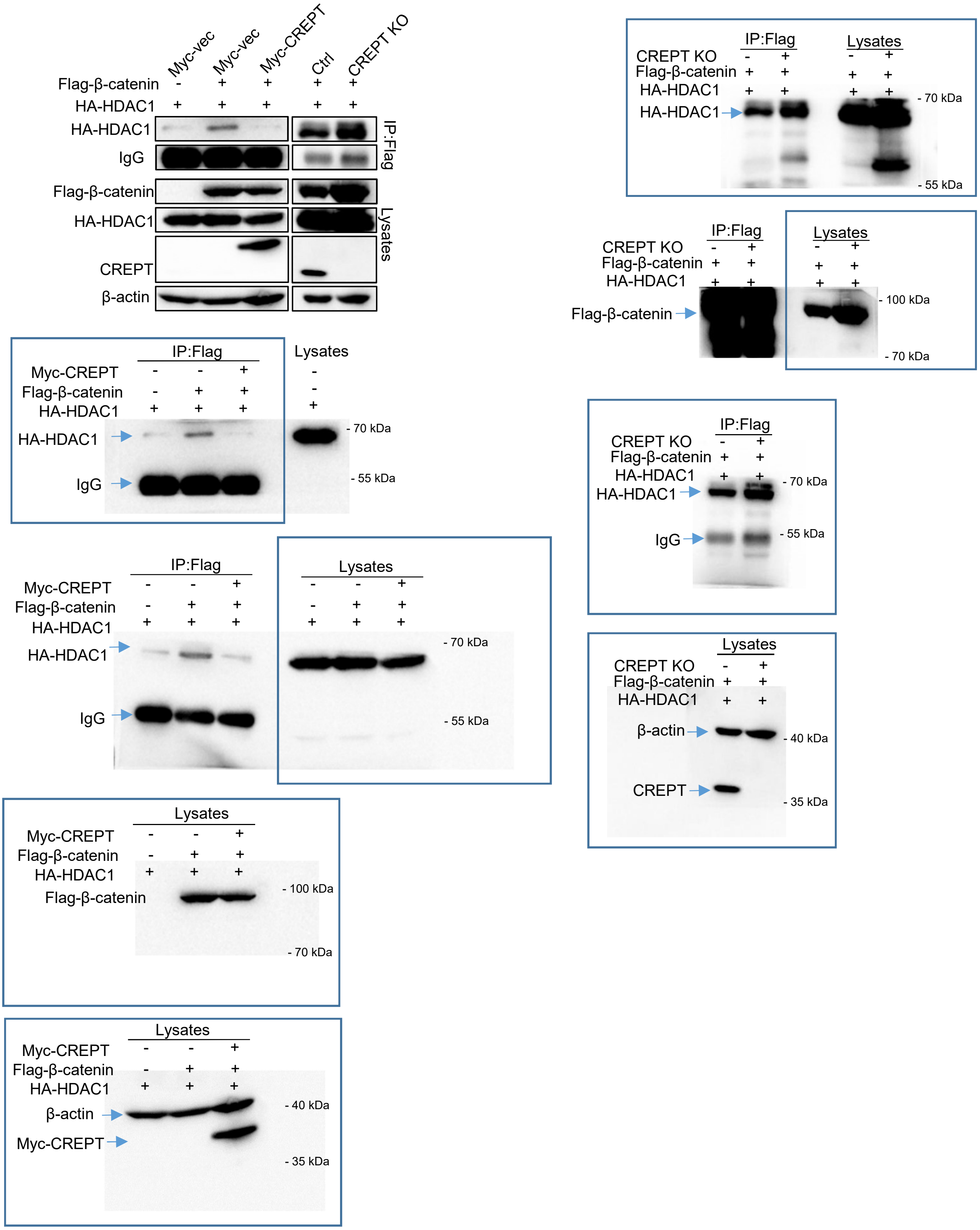
Myc-vec
Myc-vec
Myc-CREPT
Ctrl
CREPT KO
Flag-β-catenin - + + + +
HA-HDAC1 + + + + +
HA-HDAC1
IgG
IP:Flag
Flag-β-catenin
HA-HDAC1
CREPT
β-actin
Lysates
IP:Flag
Lysates
CREPT KO - + - +
Flag-β-catenin + + + +
HA-HDAC1 + + + +
HA-HDAC1
- 70 kDa
- 55 kDa
IP:Flag
Lysates
CREPT KO - + - +
Flag-β-catenin + + + +
HA-HDAC1 + + + +
Flag-β-catenin
- 100 kDa
- 70 kDa
IP:Flag
Lysates
Myc-CREPT - - + -
Flag-β-catenin - + + -
HA-HDAC1 + + + +
HA-HDAC1
IgG
- 70 kDa
- 55 kDa
IP:Flag
CREPT KO - +
Flag-β-catenin + +
HA-HDAC1 + +
HA-HDAC1
IgG
- 70 kDa
- 55 kDa
IP:Flag
Lysates
Myc-CREPT - - + - - +
Flag-β-catenin - + + - + +
HA-HDAC1 + + + + + +
HA-HDAC1
IgG
- 70 kDa
- 55 kDa
Lysates
CREPT KO - +
Flag-β-catenin + +
HA-HDAC1 + +
β-actin
CREPT
- 40 kDa
- 35 kDa
Lysates
Myc-CREPT - - +
Flag-β-catenin - + +
HA-HDAC1 + + +
Flag-β-catenin
- 100 kDa
- 70 kDa
Lysates
Myc-CREPT - - +
Flag-β-catenin - + +
HA-HDAC1 + + +
β-actin
Myc-CREPT
- 40 kDa
- 35 kDa

Figure S4G

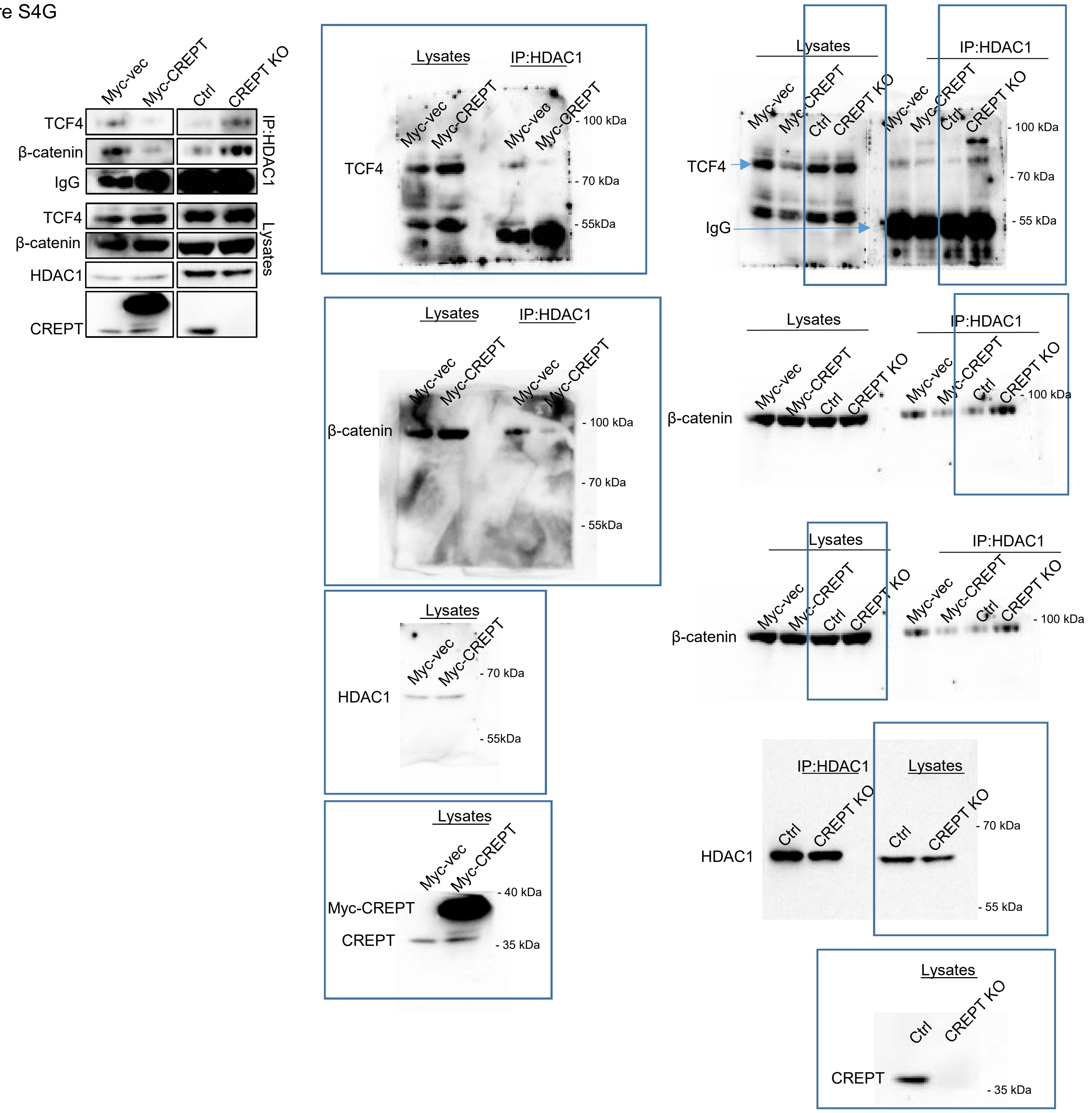
Myc-vec
Myc-CREPT
Ctrl
CREPT KO
TCF4
β-catenin
IgG
IP:HDAC1
HDAC1
CREPT
Lysates
Myc-CREPT
- 100 kDa
- 70 kDa
- 55kDa
- 55 kDa
- 40 kDa
- 35 kDa

Figure S4H

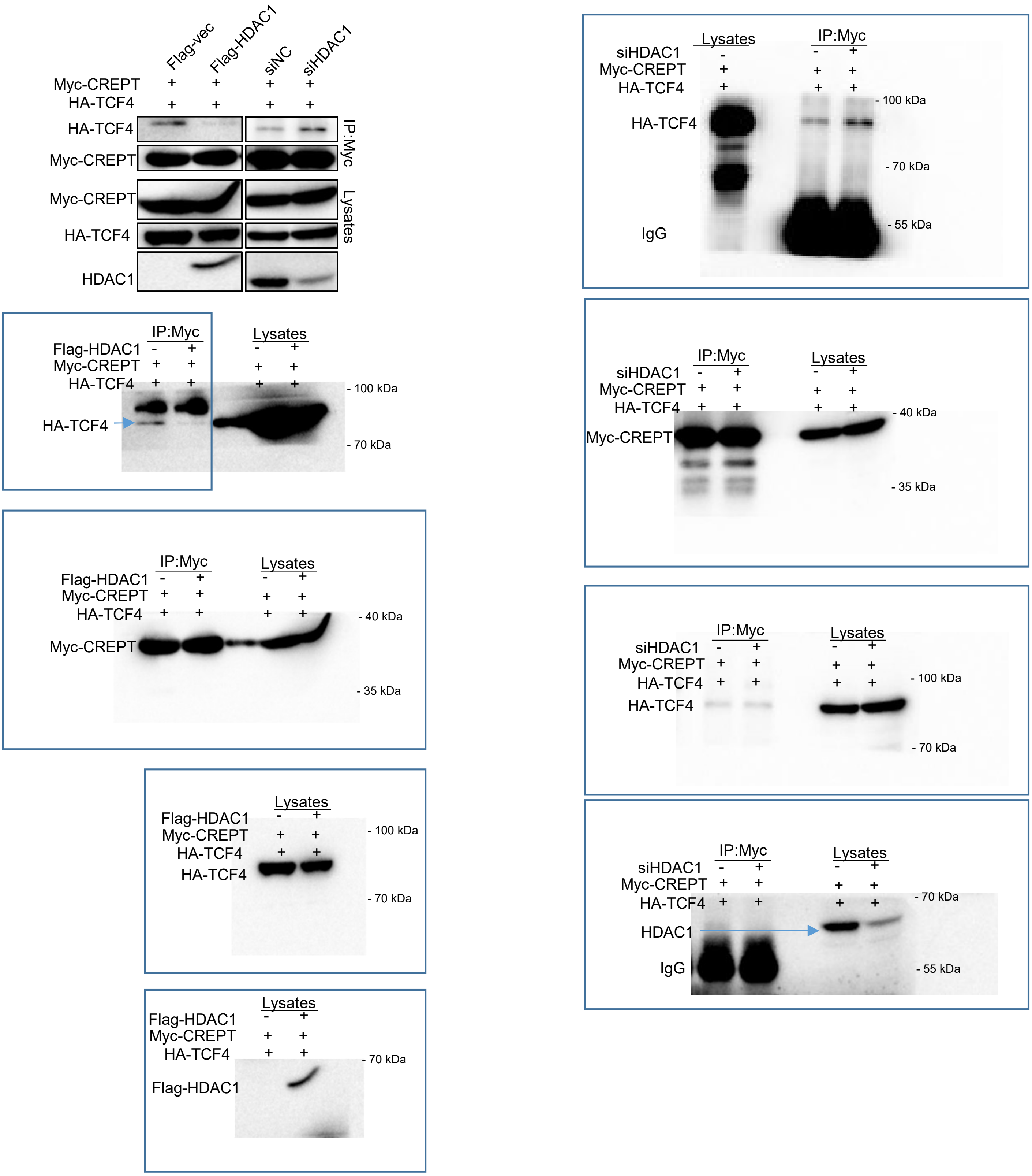

Flag-vec
Flag-HDAC1
siNC
siHDAC1
Myc-CREPT
HA-TCF4
HA-TCF4
Myc-CREPT
IP:Myc
Myc-CREPT
HA-TCF4
HDAC1
Lysates
IP:Myc
Lysates
Flag-HDAC1
Myc-CREPT
HA-TCF4
HA-TCF4
- 100 kDa
- 70 kDa
IP:Myc
Lysates
Flag-HDAC1
Myc-CREPT
HA-TCF4
Myc-CREPT
- 40 kDa
- 35 kDa
Lysates
Flag-HDAC1
Myc-CREPT
HA-TCF4
HA-TCF4
- 100 kDa
- 70 kDa
Lysates
Flag-HDAC1
Myc-CREPT
HA-TCF4
Flag-HDAC1
- 70 kDa
Lysates
IP:Myc
siHDAC1
Myc-CREPT
HA-TCF4
HA-TCF4
IgG
- 100 kDa
- 70 kDa
- 55 kDa
IP:Myc
Lysates
siHDAC1
Myc-CREPT
HA-TCF4
Myc-CREPT
- 40 kDa
- 35 kDa
IP:Myc
Lysates
siHDAC1
Myc-CREPT
HA-TCF4
HA-TCF4
- 100 kDa
- 70 kDa
IP:Myc
Lysates
siHDAC1
Myc-CREPT
HA-TCF4
HDAC1
IgG
- 70 kDa
- 55 kDa

Figure S4I

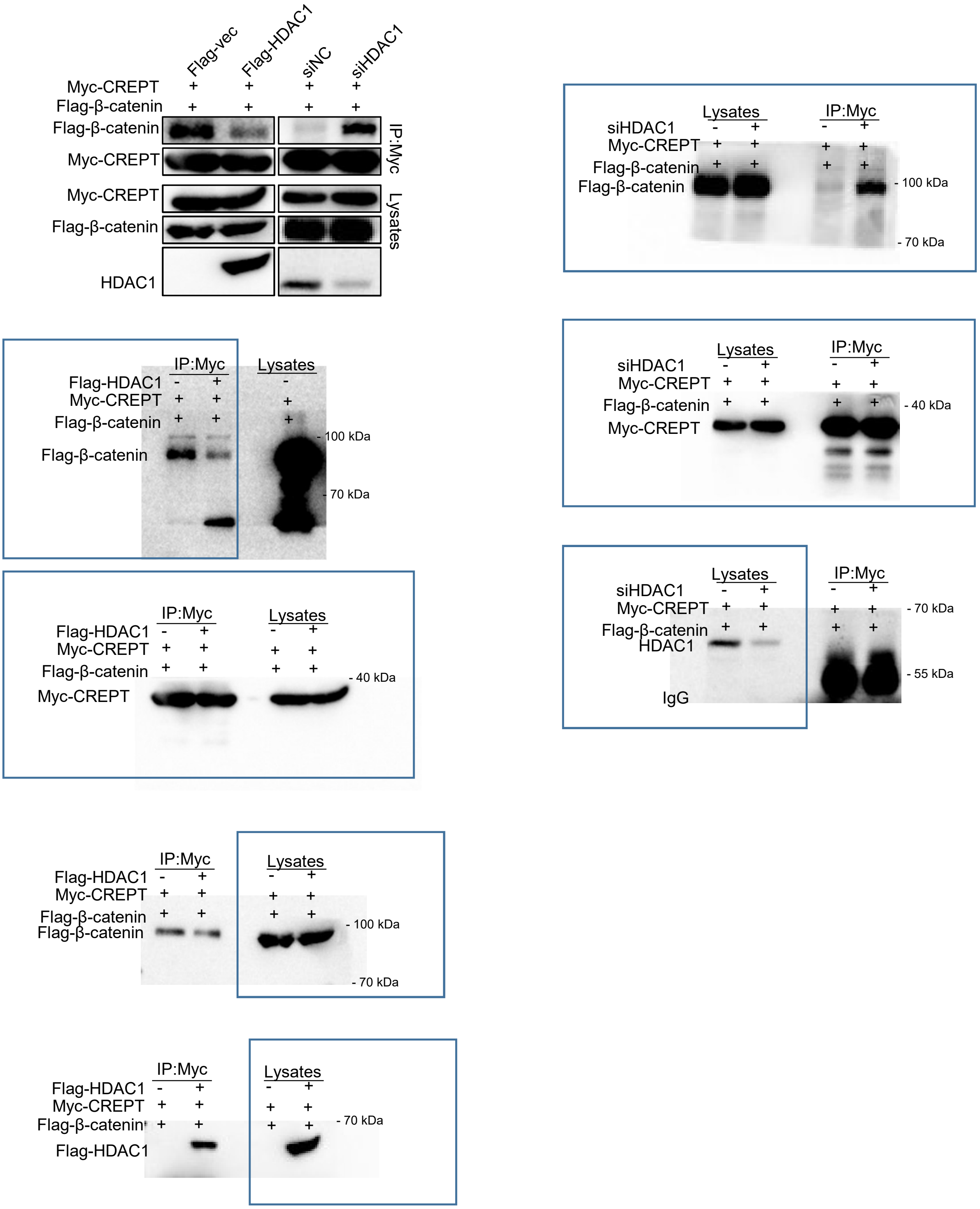

Flag-vec
Flag-HDAC1
siNC
siHDAC1
Myc-CREPT + + + +
Flag-β-catenin + + + +
Flag-β-catenin
Myc-CREPT
IP:Myc
Myc-CREPT
Flag-β-catenin
HDAC1
Lysates
Lysates
IP:Myc
siHDAC1 - + - +
Myc-CREPT + + + +
Flag-β-catenin + + + +
Flag-β-catenin
- 100 kDa
- 70 kDa
IP:Myc
Lysates
Flag-HDAC1 - + -
Myc-CREPT + + +
Flag-β-catenin + + +
Flag-β-catenin
100 kDa
70 kDa
Lysates
IP:Myc
siHDAC1 - + - +
Myc-CREPT + + + +
Flag-β-catenin + + + +
Myc-CREPT
- 40 kDa
IP:Myc
Lysates
Flag-HDAC1 - + - +
Myc-CREPT + + + +
Flag-β-catenin + + + +
Myc-CREPT
- 40 kDa
Lysates
IP:Myc
siHDAC1 - + - +
Myc-CREPT + + + +
Flag-β-catenin + + + +
HDAC1
IgG
- 70 kDa
- 55 kDa
IP:Myc
Lysates
Flag-HDAC1 - + - +
Myc-CREPT + + + +
Flag-β-catenin + + + +
Flag-β-catenin
- 100 kDa
- 70 kDa
IP:Myc
Lysates
Flag-HDAC1 - + - +
Myc-CREPT + + + +
Flag-β-catenin + + + +
Flag-HDAC1
- 70 kDa

Figure S4J

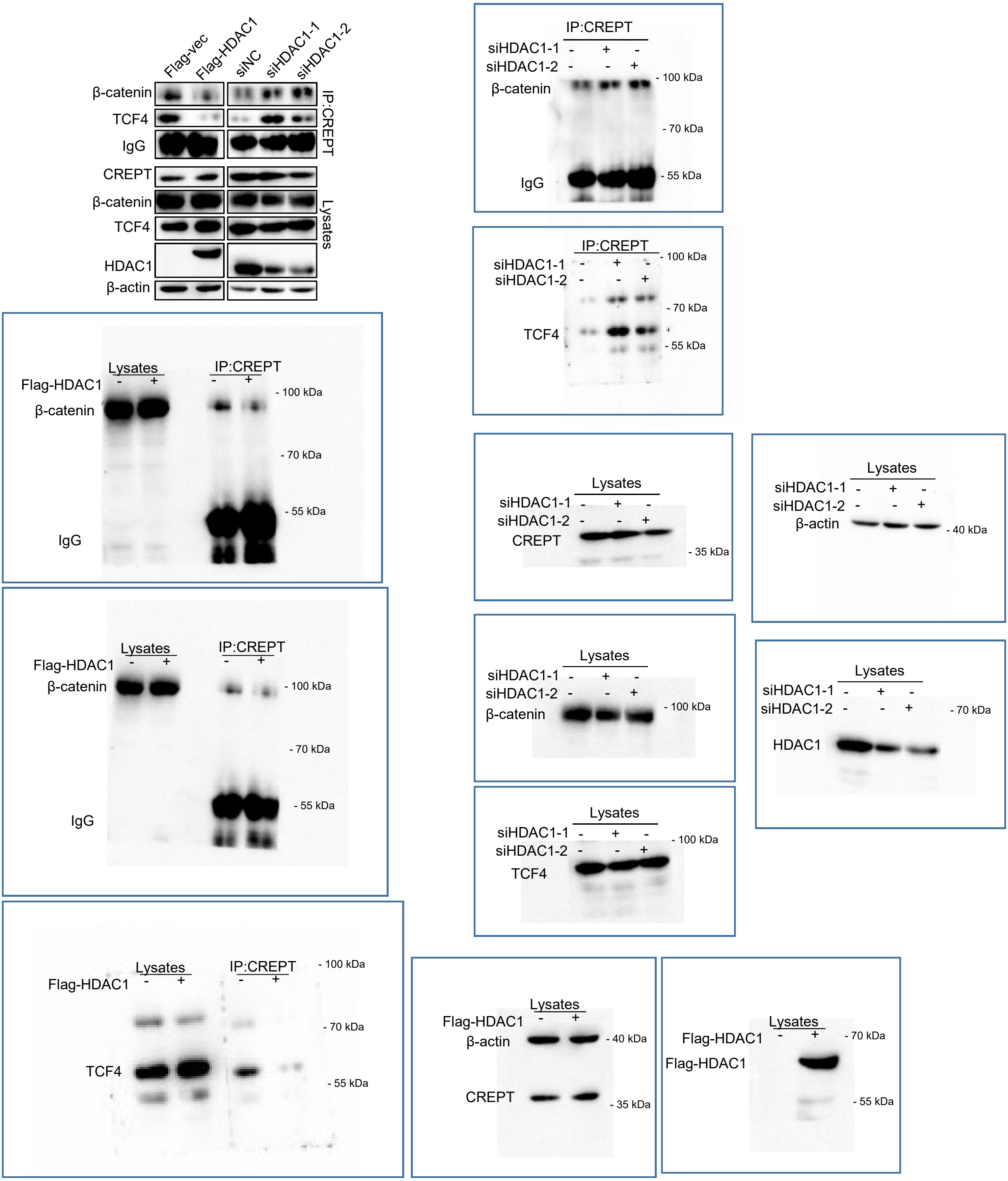

Flag-vec
Flag-HDAC1
siNC
siHDAC1-1
siHDAC1-2
β-catenin
TCF4
IgG
IP:CREPT
CREPT
β-catenin
TCF4
HDAC1
β-actin
Lysates
IP:CREPT
siHDAC1-1 - + -
siHDAC1-2 - - +
β-catenin
IgG
- 100 kDa
- 70 kDa
- 55 kDa
IP:CREPT
siHDAC1-1 - + -
siHDAC1-2 - - +
TCF4
- 100 kDa
- 70 kDa
- 55 kDa
Lysates
IP:CREPT
Flag-HDAC1 - + - +
β-catenin
IgG
- 100 kDa
- 70 kDa
- 55 kDa
Lysates
siHDAC1-1 - + -
siHDAC1-2 - - +
CREPT
- 35 kDa
Lysates
siHDAC1-1 - + -
siHDAC1-2 - - +
β-actin
- 40 kDa
Lysates
IP:CREPT
Flag-HDAC1 - + - +
β-catenin
IgG
- 100 kDa
- 70 kDa
- 55 kDa
Lysates
siHDAC1-1 - + -
siHDAC1-2 - - +
β-catenin
- 100 kDa
Lysates
siHDAC1-1 - + -
siHDAC1-2 - - +
HDAC1
- 70 kDa
Lysates
siHDAC1-1 - + -
siHDAC1-2 - - +
TCF4
- 100 kDa
Lysates
IP:CREPT
Flag-HDAC1 - + - +
TCF4
- 100 kDa
- 70 kDa
- 55 kDa
Lysates
Flag-HDAC1 - +
β-actin
CREPT
- 40 kDa
- 35 kDa
Lysates
Flag-HDAC1 - +
Flag-HDAC1
- 70 kDa
- 55 kDa

Figure S4K

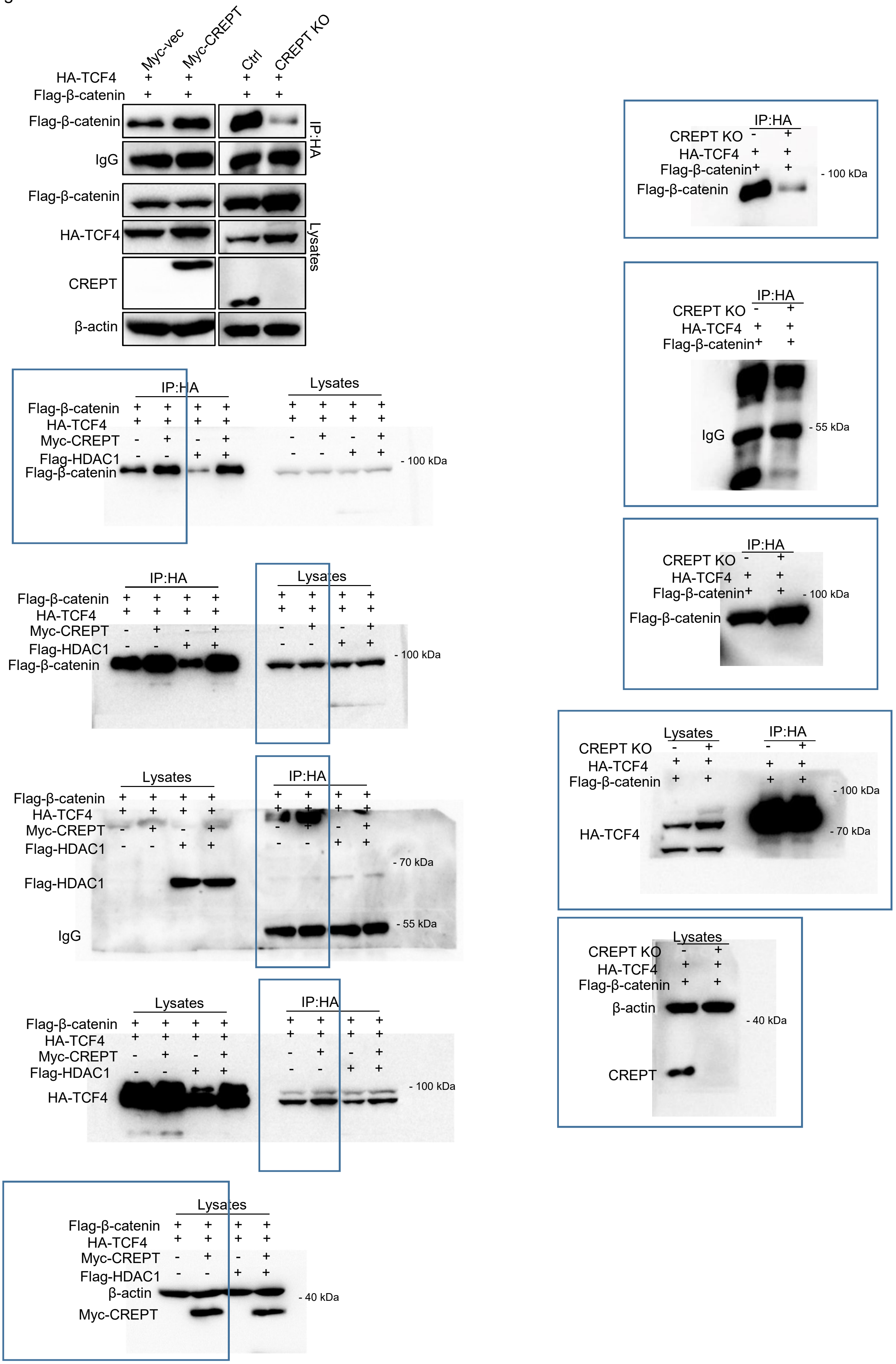

Myc-vec
Myc-CREPT
Ctrl
CREPT KO
HA-TCF4
Flag-β-catenin
Flag-β-catenin
IgG
IP:HA
Flag-β-catenin
HA-TCF4
CREPT
β-actin
Lysates
IP:HA
CREPT KO
HA-TCF4
Flag-β-catenin
Flag-β-catenin
- 100 kDa
IP:HA
Lysates
Flag-β-catenin
HA-TCF4
Myc-CREPT
Flag-HDAC1
Flag-β-catenin
- 100 kDa
IP:HA
CREPT KO
HA-TCF4
Flag-β-catenin
IgG
- 55 kDa
IP:HA
CREPT KO
HA-TCF4
Flag-β-catenin
Flag-β-catenin
- 100 kDa
IP:HA
Lysates
Flag-β-catenin
HA-TCF4
Myc-CREPT
Flag-HDAC1
Flag-β-catenin
- 100 kDa
Lysates
IP:HA
CREPT KO
HA-TCF4
Flag-β-catenin
HA-TCF4
- 100 kDa
- 70 kDa
Lysates
IP:HA
Flag-β-catenin
HA-TCF4
Myc-CREPT
Flag-HDAC1
Flag-HDAC1
IgG
- 70 kDa
- 55 kDa
Lysates
CREPT KO
HA-TCF4
Flag-β-catenin
β-actin
CREPT
- 40 kDa
Lysates
IP:HA
Flag-β-catenin
HA-TCF4
Myc-CREPT
Flag-HDAC1
HA-TCF4
- 100 kDa
Lysates
Flag-β-catenin
HA-TCF4
Myc-CREPT
Flag-HDAC1
β-actin
Myc-CREPT
- 40 kDa

Figure S4L

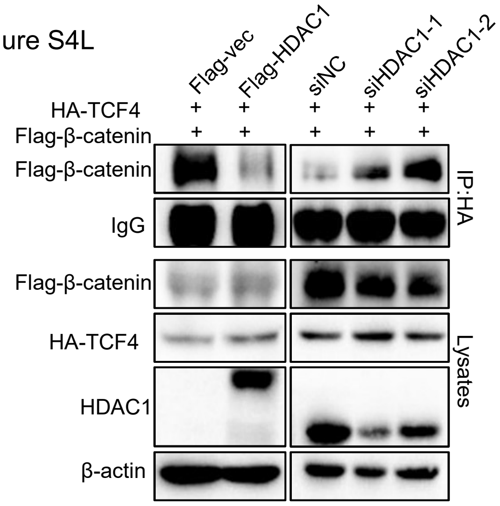
Flag-vec
Flag-HDAC1
siNC
siHDAC1-1
siHDAC1-2
HA-TCF4
Flag-β-catenin
Flag-β-catenin
IgG
IP:HA
Flag-β-catenin
HA-TCF4
HDAC1
β-actin
Lysates

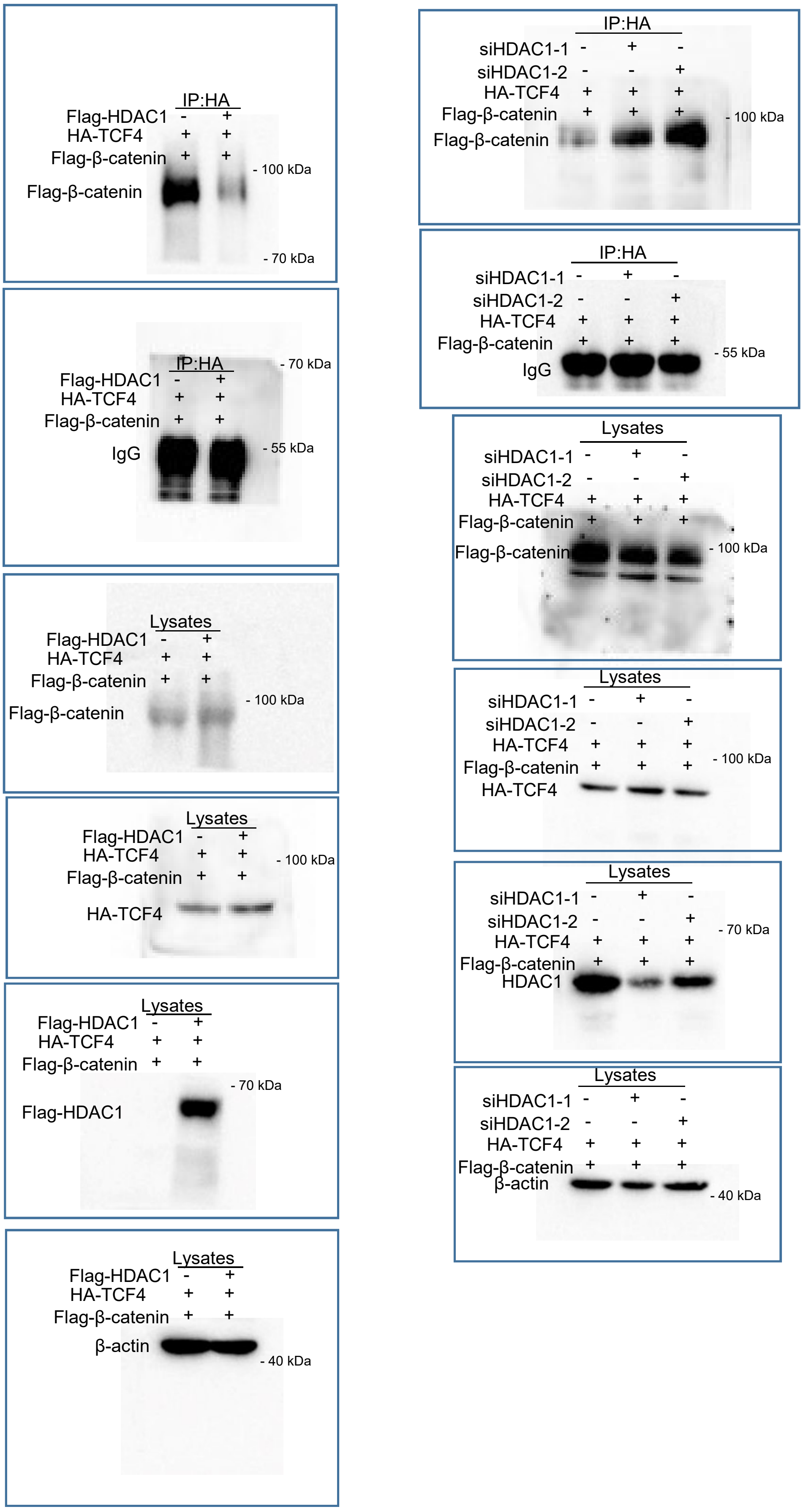
IP:HA
Flag-HDAC1
HA-TCF4
Flag-β-catenin
Flag-β-catenin
- 100 kDa
- 70 kDa
IP:HA
IgG
- 70 kDa
- 55 kDa
Lysates
Flag-β-catenin
- 100 kDa
Lysates
HA-TCF4
- 100 kDa
Lysates
Flag-HDAC1
- 70 kDa
Lysates
β-actin
- 40 kDa
IP:HA
siHDAC1-1
siHDAC1-2
HA-TCF4
Flag-β-catenin
Flag-β-catenin
- 100 kDa
IP:HA
IgG
- 55 kDa
Lysates
Flag-β-catenin
- 100 kDa
Lysates
HA-TCF4
- 100 kDa
Lysates
HDAC1
- 70 kDa
Lysates
β-actin
- 40 kDa

Figure S4M

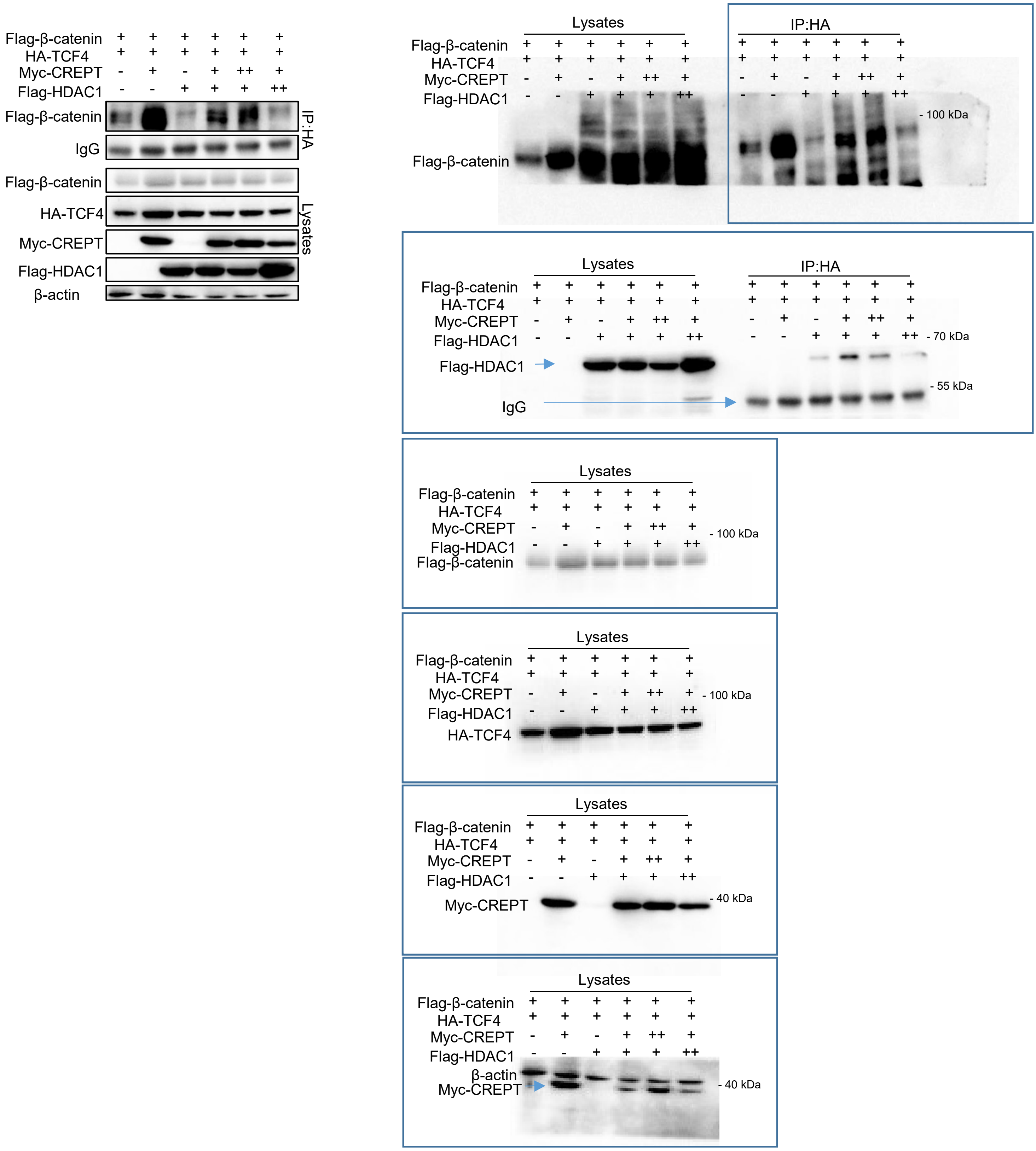

Flag-β-catenin
HA-TCF4
Myc-CREPT
Flag-HDAC1
IgG
β-actin
IP:HA
Lysates
- 100 kDa
- 70 kDa
- 55 kDa
- 40 kDa

Figure S6A

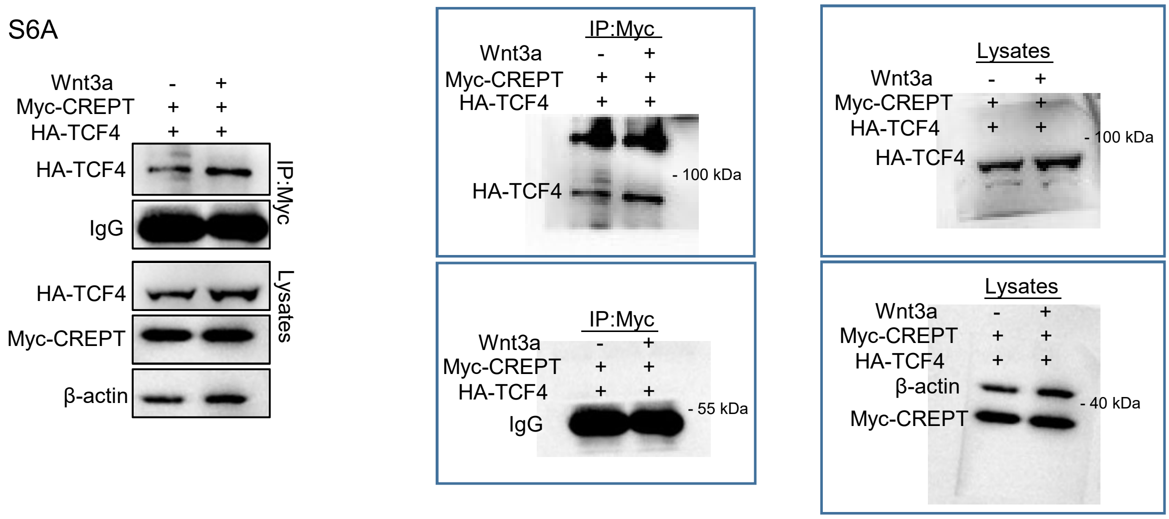

Figure S6B

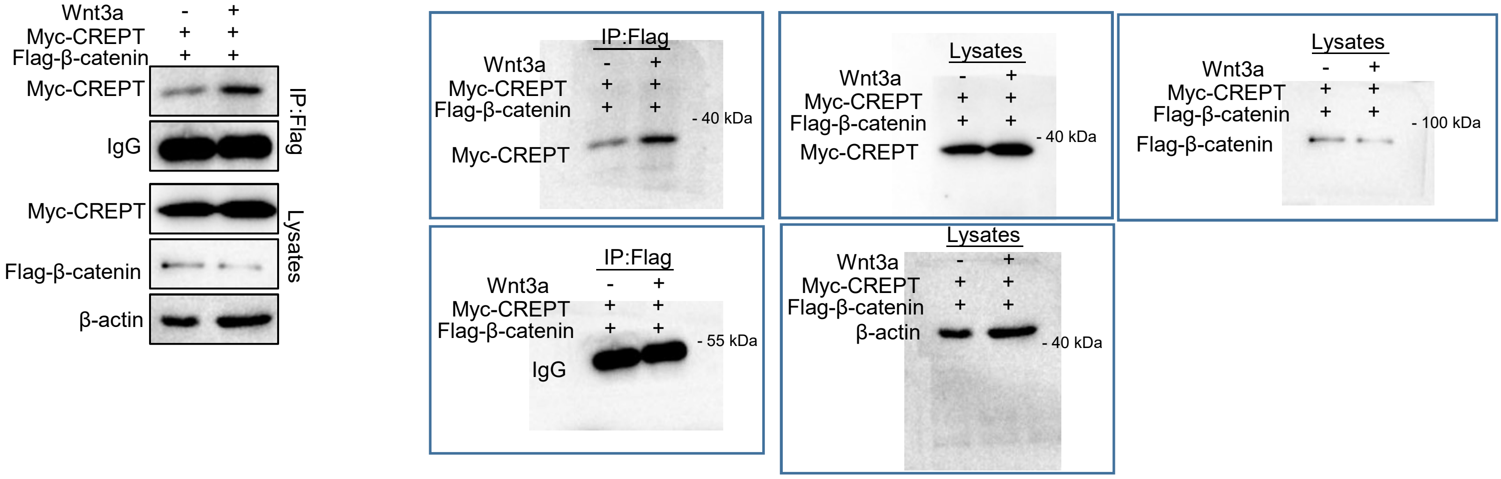

Figure S6C

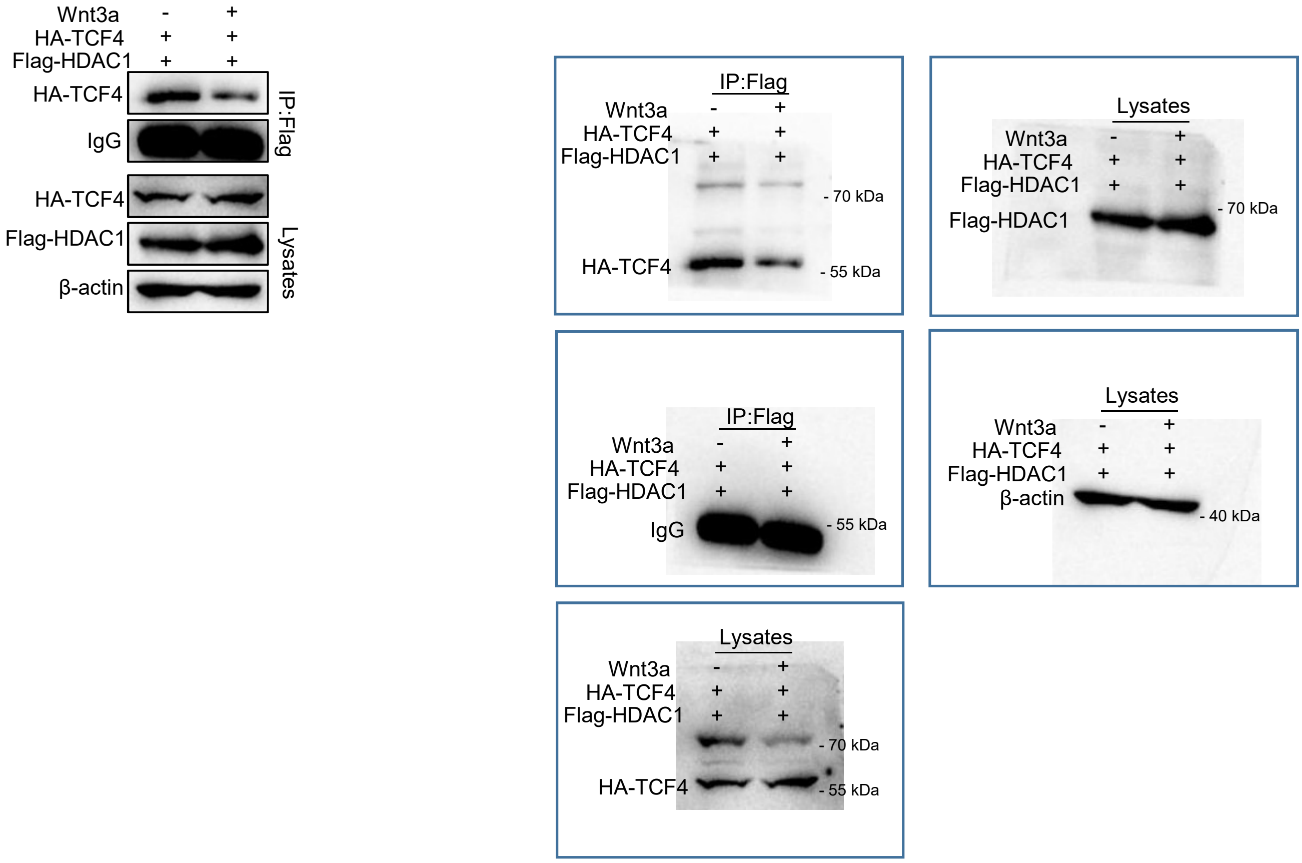

Figure S6D

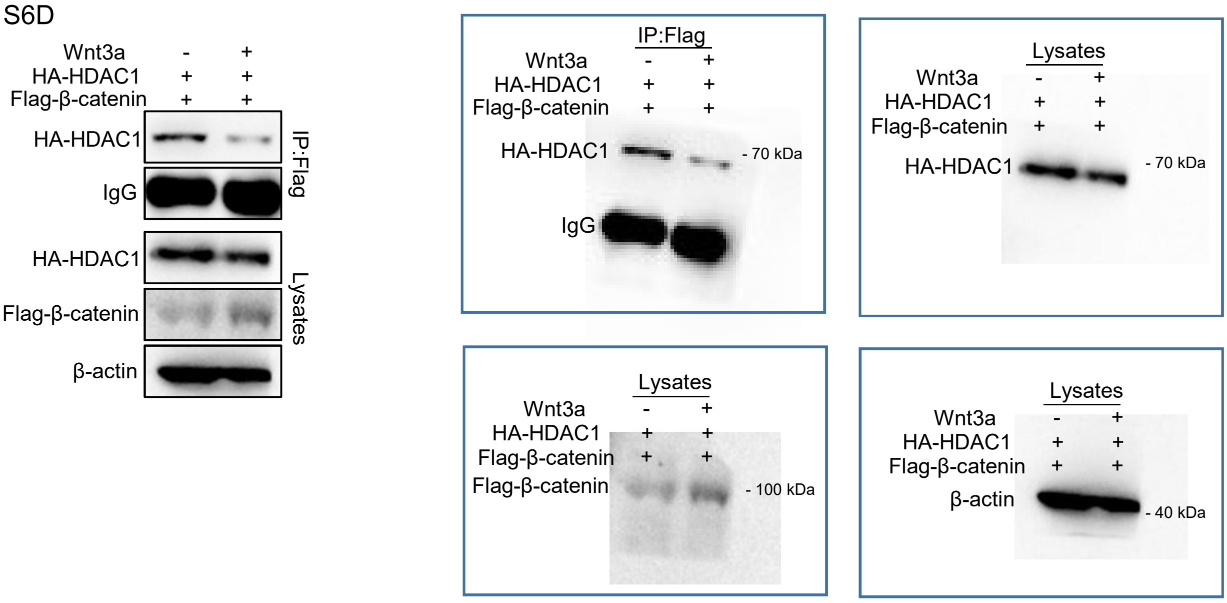

Figure S6E

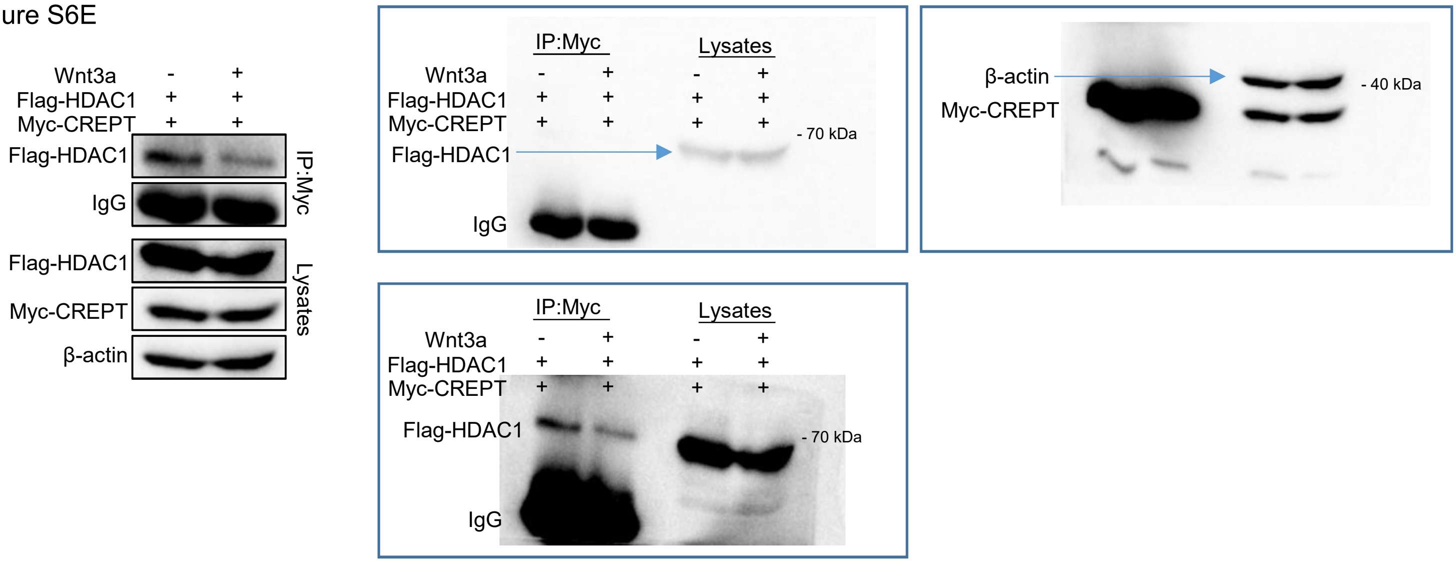

Figure S6F

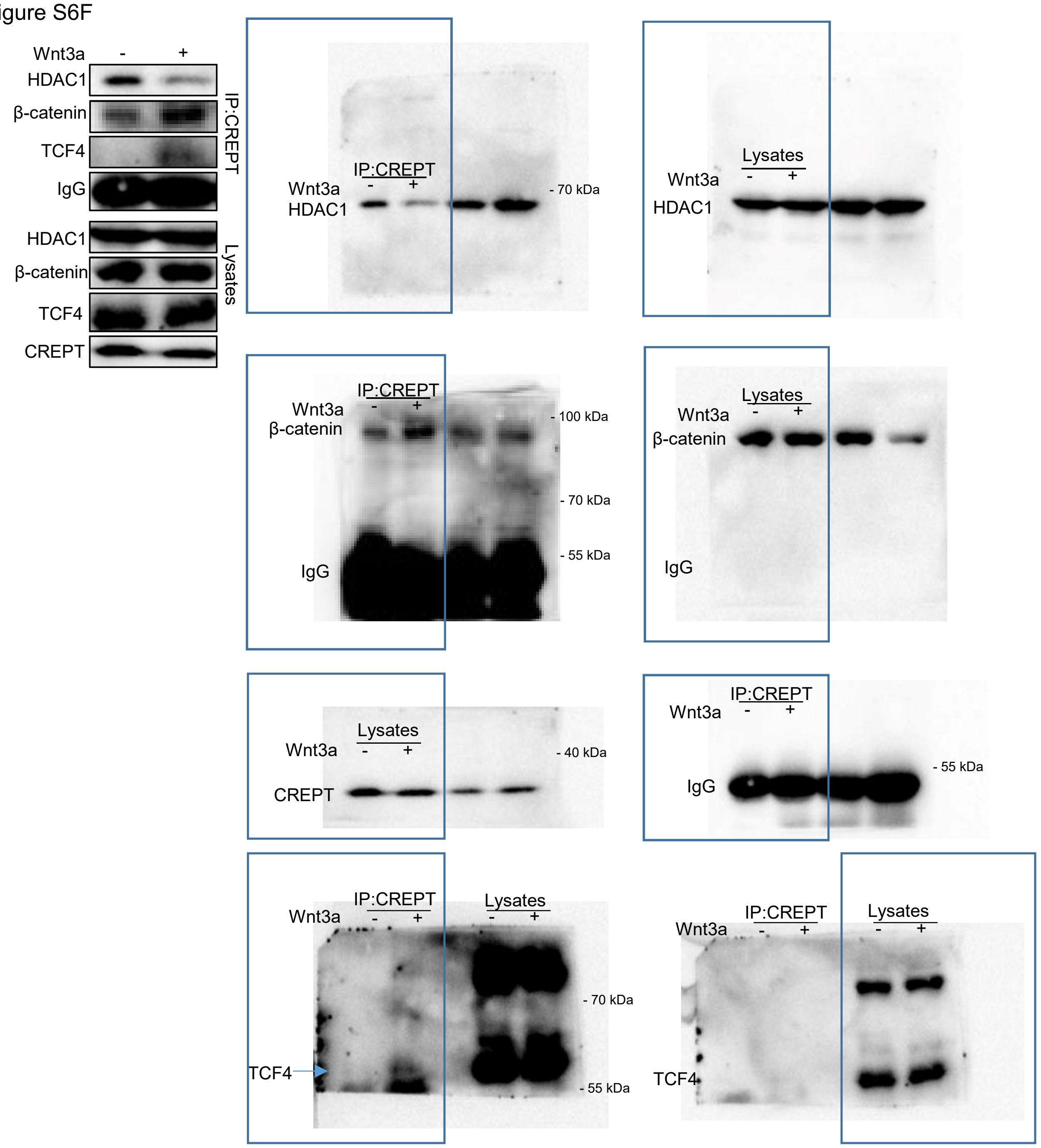

Wnt3a
-
+
HDAC1
β-catenin
TCF4
IgG
IP:CREPT
HDAC1
β-catenin
TCF4
CREPT
Lysates
IP:CREPT
Wnt3a
HDAC1
- 70 kDa
Lysates
Wnt3a
HDAC1
IP:CREPT
Wnt3a
β-catenin
- 100 kDa
- 70 kDa
- 55 kDa
IgG
Lysates
Wnt3a
β-catenin
IgG
Lysates
Wnt3a
CREPT
- 40 kDa
IP:CREPT
Wnt3a
IgG
- 55 kDa
IP:CREPT
Lysates
Wnt3a
TCF4
- 70 kDa
- 55 kDa
IP:CREPT
Lysates
Wnt3a
TCF4
